# Short-read aligner performance in germline variant identification

**DOI:** 10.1093/bioinformatics/btad480

**Published:** 2023-08-01

**Authors:** Richard Wilton, Alexander S Szalay

**Affiliations:** Department of Physics and Astronomy, Johns Hopkins University, Baltimore, MD 21218, United States; Department of Physics and Astronomy, Johns Hopkins University, Baltimore, MD 21218, United States; Department of Computer Science, Johns Hopkins University, Baltimore, MD 21218, United States

## Abstract

**Motivation:**

Read alignment is an essential first step in the characterization of DNA sequence variation. The accuracy of variant-calling results depends not only on the quality of read alignment and variant-calling software but also on the interaction between these complex software tools.

**Results:**

In this review, we evaluate short-read aligner performance with the goal of optimizing germline variant-calling accuracy. We examine the performance of three general-purpose short-read aligners—BWA-MEM, Bowtie 2, and Arioc—in conjunction with three germline variant callers: DeepVariant, FreeBayes, and GATK HaplotypeCaller. We discuss the behavior of the read aligners with regard to the data elements on which the variant callers rely, and illustrate how the runtime configurations of these software tools combine to affect variant-calling performance.

## 1 Introduction

Short DNA sequencer reads are widely used to identify short variants—single-nucleotide polymorphisms (SNPs) as well as short (fewer than ∼20 bp) insertions and deletions in whole-genome sequencing (WGS) samples. The fundamental first step in characterizing sequence variants in short reads is to use alignment software to map the reads to the locations in a reference genome where their base sequences are the most similar. The mappings and error probabilities produced by an aligner can be analyzed by a variety of variant-calling software tools to characterize the locations and genotypes of SNPs and indels in WGS samples.

The input to a variant caller is always the output from a read aligner, so a variant caller’s performance inevitably depends on that of the read aligner with which it is coupled. For this reason, it is important to evaluate the performance of both the variant caller and the aligner in the context of how these complex software tools interact with each other.

### 1.1 A note on terminology

In this article, we use *alignment* to refer to the computation of the similarity between a read sequence and a reference sequence; we use *mapping* to indicate the result of an alignment computation. We also use *sequence* not in an algorithmic sense but simply to refer to an ordered chain of nucleotide bases.

### 1.2 Evaluating variant caller performance

There are two complementary aspects to measuring variant caller performance. Read aligners and variant callers produce metadata that represent the error probabilities associated with read mappings and with variant calls. These metrics are estimated from the characteristics of the aligned read sequence data. In addition, the accuracy of variant identification can be measured with respect to benchmark collections of variants whose locations and genotypes are confidently identified within a specific reference genome. Such benchmarks make it possible to compare experimental variant calls with a trustworthy list of known variants in a specified region (or set of regions) in a reference genome.

#### 1.2.1 Data formats

Short-read aligners associate performance-related metadata with read mappings in SAM- or BAM-formatted output data files (SAM/BAM Format Specification Working Group 2022). Variant callers do this in VCF-formatted output (Global Alliance for Genomics and Health 2022). Table 1 summarizes the salient data items in these file formats.

**Table 1.** Data fields useful for performance evaluation in short-read aligner output (SAM/BAM format) and variant caller output (VCF format).

**SAM/BAM**	
RNAME	reference subunit name (e.g. “chr1”)
POS	reference sequence location of first mapped base
MAPQ	probability that the mapping location (RNAME, POS) is wrong
TLEN	number of reference sequence bases spanned by paired-end mapping
SEQ	read base sequence
QUAL	base quality scores (BQS); per-base probability of sequencer error
AS	alignment score
**VCF**	
CHROM	reference subunit name (e.g. “chr1”)
POS	reference sequence location of first variant base
REF	reference base sequence (with regard to variant)
ALT	variant base sequence
QUAL	probability that the variant call in ALT is wrong
INFO.QD	QUAL normalized by read depth
FORMAT.DP	read depth (number of reads that support the variant call)
FORMAT.GQ	per-genotype probability that the genotype call is wrong

*Note:* In VCF format, QD is a subfield of the INFO field; DP and GQ are per-sample subfields defined in the FORMAT field.

#### 1.2.2 Quality values

The term *quality* is used in both SAM/BAM and VCF formats to indicate an error probability estimated by the aligner or variant caller implementation. In these formats, a quality value (MAPQ, BQS, QUAL, GQ) is represented as a logarithmic transformation of an error probability P_e_:


$$-10\times {\text{log}\;}_{10}\left(P_{e}\right).$$

This transformation associates higher quality values with lower error probabilities.

#### 1.2.3 Aligner quality metrics

Short-read aligners report two essential quality metrics. Base quality scores (BQS) are estimated by the read sequencer and reported unchanged by the aligner. Mapping quality (MAPQ) represents the probability that the aligner has reported a read mapping at an incorrect position (RNAME, POS) in a reference genome. These four read-mapping metadata are essential to the discovery and characterization of potential read sequence variation.

#### 1.2.4 Variant quality metrics

Variant callers associate multiple quality estimates with each reported variant. These metrics include QUAL (the probability that the caller wrongly reported the existence of a variant), QD (QUAL normalized by the number of reads that support the assumption that variant exists at a particular location), and GQ (the probability that the genotype assigned by the caller is incorrect). Although the computational model for these metrics depends on the implementation details of the variant caller, their reported values ultimately depend on the read-mapping information produced by the read aligner.

In addition, it is possible to produce variant quality metrics by applying statistical models to the ensemble of variant calls for a set of reads. For example, the GATK VariantRecalibrator computes VQSLOD (an odds ratio of being a true variant); hap.py reports QQ (used in conjunction with ROC curves computed by the tool). This additional postprocessing may improve variant-calling accuracy by better discriminating between true positive and false positive variant calls than the metrics generated by the variant caller.

#### 1.2.5 Aligner metadata for variant discovery

Variant callers use RNAME and POS to group read sequences by reference-genome position. A potential variant exists at each reference-genome location where multiple reads have mappings in which one or more bases differ consistently from the reference at that location, and where the differing bases in the read sequence have sufficiently high BQS.

In addition, the number of reads that map across the location of a variant (“coverage” at that location) is essential to a variant caller’s computational model for QUAL and GQ probability estimates. In general, QUAL and GQ increase with greater coverage. In low coverage, “hard-to-map” regions [e.g. regions that are highly polymorphic and therefore poorly represented in reference genome sequence, or GC-rich regions susceptible to sequencing bias (Ross *et al.* 2013)], the aligner may report an insufficient number of reads for the variant caller to identify variants. Also, excessive coverage in a region (e.g. due to copy number variation) may be associated with false-positive heterozygous genotype calls (Li 2014a, Kishikawa *et al.* 2019).

#### 1.2.6 Alignment quality influences variant quality

The relationship between coverage and variant-call quality assumes that the read aligner reports mappings in the correct location (RNAME, POS) in the reference genome. However, there is an error probability associated with each read’s reported mapping location. Read aligners report this error probability as MAPQ.

Variant callers use MAPQ as a constraint on the identification of potential variants by supporting a minimum MAPQ threshold value that excludes below-threshold mappings from further analysis. The threshold may be configured to any reasonable value. For example, a MAPQ threshold of 4 excludes reads that have equivalent mappings in two or more different reference-genome locations. (A read aligner must always report MAPQ ≤ 3 for such reads because the probability that they are mapped in the wrong location is at least 50%.) The GATK HaplotypeCaller uses a default MAPQ threshold of 20 (i.e. the probability that a mapping is incorrectly placed is 1%).

MAPQ may also influence a variant caller’s computation of variant quality probabilities. For example, GQ tends to increase with higher MAPQ in the supporting reads (Supplementary Fig. SF2), especially with MAPQ near the low end of its usable range.

**Table:** 

recall: $\frac{{\mathrm{TP}}_{T}}{\left({\mathrm{TP}}_{T}+{\mathrm{FN}}_{T}\right)}$	TP_*T*_: TP calls in the truth set FN_*T*_: FN calls in the truth set
precision: $\frac{{\mathrm{TP}}_{Q}}{\left({\mathrm{TP}}_{Q}+{\mathrm{FP}}_{Q}\right)}$	TP_*Q*_: TP calls in the query set FP_*Q*_: FP calls in the query set
F1: $\frac{2\times \mathrm{recall}\times \mathrm{precision}}{\left(\mathrm{recall}+\mathrm{precision}\right)}$	*F*-measure (harmonic mean of recall and precision)

#### 1.2.7 Benchmark comparisons

Variant-calling accuracy is quantified by comparing the variants reported by a variant caller (the “query set”) to a list of trusted, well-characterized variants in a reference genome (a “truth set”). Accuracy can be characterized by accumulating counts of true and false positive (TP, FP) calls as well as true and false negative (TN, FN) calls, where a TP call is reported with the same genotype and location as a known variant. This kind of benchmark analysis can be performed with a tool such as hap.py (Krusche 2021), which produces a variety of aggregate counts and summary statistics, including the following:

With paired-end Illumina sequencer reads aligned to the human reference genome, toolchains that use the aligners and variant callers discussed here can routinely call benchmark variants with ∼0.99 recall and precision in WGS samples, with ∼0.96 recall and precision in hard-to-align repetitive and highly variable regions (Wagner *et al.* 2022).

These metrics can be corroborated by observing the ratio of transitions to transversions (*T*_i_/*T*_v_) in SNPs, which is expected to be approximately 2 in human WGS samples (Halushka *et al.* 1999, Venter *et al.* 2001, McKernan *et al.* 2009). The overall *T*_i_/*T*_v_ ratio decreases in the presence of randomly occurring variant-calling errors (i.e. errors where transitions and transversions are equally likely).

### 1.3 Evaluating short-read aligner performance in variant calling

The goal of performance optimization in variant analysis is to obtain accurate variant-calling results, but variant-calling results *per se* depend on the configurations of both the read aligner and the variant caller. Although default configurations usually produce adequate results, optimization always requires consideration of specific variant analysis goals as well as the characteristics of the reference genome and the read mappings being analyzed. Each of the variant callers we discuss here supports dozens of runtime parameters through which performance can be adapted to specific analysis requirements.

#### 1.3.1 Analysis toolchain

With this in mind, we focused on short-read aligner performance by using a variant analysis toolchain that contained no caller-specific optimizations. In this way, variant-calling accuracy could be correlated to short-read aligner configuration, even if performance for each individual caller (or combination of aligner and caller) might have been improved by such optimizations:

- short-read alignment:
  ○ BWA-MEM2 (Vasimuddin *et al.* 2019)
  ○ Bowtie 2 (Langmead and Salzberg 2012)
  ○ Arioc (Wilton *et al.* 2015)
- filter mappings (proper mappings having MAPQ ≥ 4):
  ○ samtools (Danecek *et al.* 2021)
- remove duplicate mappings:
  ○ GATK (Poplin *et al.* 2018): MarkDuplicates
- identify variants:
  ○ GATK: HaplotypeCaller, GenotypeGVCFs
  ○ FreeBayes (Garrison and Marth 2012)
  ○ DeepVariant (Poplin *et al.* 2018)
- separate SNPs from indels and other (“mixed”) variants:
  ○ GATK: SelectVariants
- identify optimal variant hard-filtering criteria (maximize F1):
  ○ vcfeval (Cleary *et al.* 2015)
- filter SNPs and INDELs separately using variant caller metadata:
  ○ GATK: VariantFiltration
- aggregate variant-call results:
  ○ hap.py

We evaluated three general-purpose short-read aligners (BWA-MEM, Bowtie 2, and Arioc) and three short-variant callers (GATK HaplotypeCaller, FreeBayes, and DeepVariant) whose accuracy and speed have been established. Each of these tools supports runtime configuration options that control a variety of computational behaviors, and each produces a full set of descriptive metadata that can be used to measure performance.

#### 1.3.2 Reference benchmarks

With each of these software tools, we characterized the variants in Illumina paired-end reads from GIAB sample HG002 (Zook *et al.* 2019, NIST 2020) using v4.2.1 of the benchmark annotations produced by the GIAB Consortium and the GA4GH Benchmarking Team (NIST 2022) for SNP and short indel variants in GRCh37 and GRCh38 (Olson *et al.* 2022, Wagner *et al.* 2022). These benchmark data include reliably identified variants in reference genome regions with a high prevalence of repetitive sequence or with high variability; such regions are typically mapped with low coverage by general-purpose short-read aligners.

Read-mapping accuracy in such “hard-to-map” regions is a stringent test of the ability of a short-read aligner to discover maximum similarity mappings for reads that do not have perfect or near-perfect mappings, and variant discovery in these regions tests the ability of a variant caller to discriminate among potential variant calls where read coverage and mappings do not unambiguously support only one possible call.

#### 1.3.3 Tool-oblivious filtering

To avoid inadvertent bias toward any particular aligner or variant caller, our analysis toolchain incorporated only minimal filtering of read mappings and variant calls. We used read-mapping filters only to limit analysis to unduplicated proper mappings having MAPQ ≥ 4; we filtered variant calls on QUAL, QD, and MQ using thresholds associated with maximal F1 (*F*-measure) as reported by vcfeval.

## 2 Paired-end mapping topology affects variant-calling accuracy

In short-read alignment, longer read sequences are more likely to have unique mapping locations than shorter reads (Palmieri and Schlötterer 2009). Short-read aligners also obtain better overall mapping-location accuracy with paired-end reads that result from sequencing DNA fragments that are hundreds of bases long (Li *et al.* 2008) than with unpaired reads.

This increased read-mapping accuracy occurs with reads that map into multiple repetitive reference genome regions that are highly similar but not identical to each other. It also occurs when read mappings contain short regions of high variability, since the aligner can only place such a read accurately in the reference genome when most of the read sequence maps with high similarity to adjacent, low-variability regions.

### 2.1 Paired-end mapping increases sensitivity

In both cases, higher read-mapping accuracy should imply higher variant-calling accuracy. Nevertheless, optimal use of paired-end mappings requires appropriate configuration of both the read aligner and the variant caller.

#### 2.1.1 Repetitive regions

Significant proportions of all prokaryotic and eukaryotic genomes are repetitive (Haubold and Wiehe 2006). At least 50% and up to 69% (de Koning *et al.* 2011) of the human reference genome consists of repetitive elements, including tandem repeats, transposable elements, and multi-kilobase segmental duplications that are at least 98% similar to other regions in the genome (Bailey *et al.* 2002). Reads that map within such regions tend to have multiple plausible high-scoring mappings and therefore higher associated error probabilities (lower MAPQ) associated with the placement of their mappings.

When a read sequence has equivalent high-scoring mappings at two or more reference genome locations (each associated with MAPQ ≤ 3), an aligner may report the alternative mapping locations in SAM/BAM format in different ways: by emitting multiple records, each flagged as secondary mappings; by reporting one of the possible mapping locations as a primary mapping and listing the alternative locations in an optional field; or by randomly “choosing” one mapping location to report. But regardless of how multiple possible mappings are reported for a read, significant computational analysis is still needed to disambiguate them prior to variant discovery (Prodanov and Bansal 2022).

With paired-end mappings, this problem is mitigated when only one mate maps within a repetitive region. When the opposite mate maps uniquely and with high similarity to the reference sequence, an aligner can infer that the pair’s mapping location is reliable and accordingly assign it a higher MAPQ. The read aligners evaluated here implement this heuristic explicitly when computing MAPQ for such mappings.

#### 2.1.2 Hard-to-align regions

A similar heuristic can be applied when only one mate maps in a low-similarity, hard-to-align region (e.g. highly polymorphic regions such as the MHC). When its opposite mate maps accurately and uniquely, the aligner can report a higher MAPQ for the low-similarity mate mapping. A variant caller that uses MAPQ to estimate the reliability of read mappings across potential sites of variation will then be more likely to find characterizable variation within that hard-to-align region.

### 2.2 Overlapped, covering, and dovetailed mappings

There are situations where the topology of a paired-end mapping compromises the aligner’s ability to reliably assign a mapping location. This can occur when the mate mappings overlap to some extent. The accuracy of variant calling can depend on how paired-end overlap is handled in the read aligner and in post-alignment read filtering.

**Figure 1.**
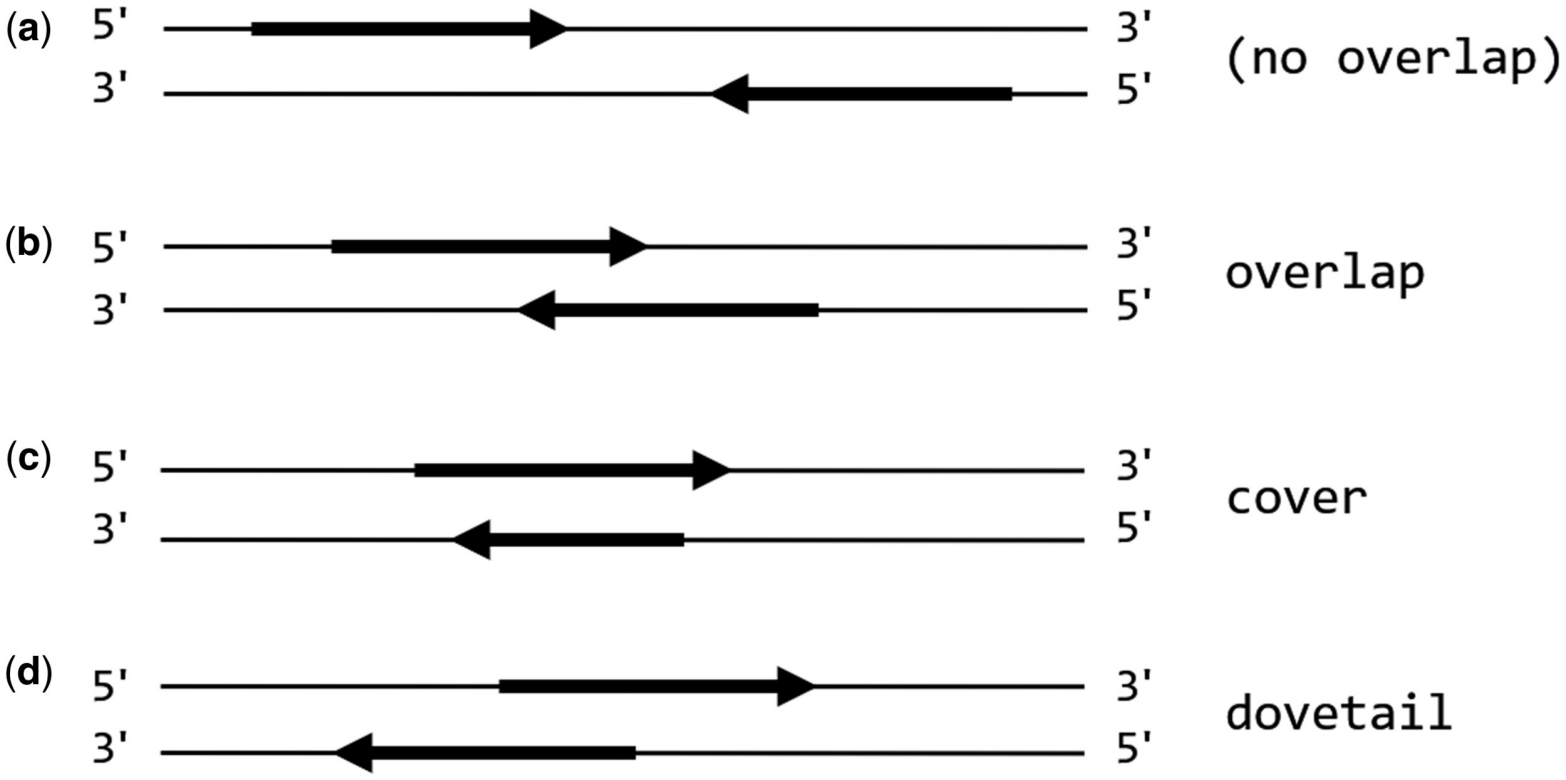
Topology of paired-end mate mappings: (a) mates map to discrete regions of the reference genome, with the forward mate “upstream” of the reverse complement mate; (b) mate mappings overlap at one or more base positions; (c) one mate maps entirely within the region covered by the opposite mate; (d) the reverse complement mate maps “upstream” of the forward mate.

Figure 1 illustrates overlap topologies for paired-end mappings produced by Illumina sequencing technology, where opposite ends of each double-stranded DNA fragment are sequenced in the 5′–3′ direction on opposite DNA strands. The expected mapping topology (Fig. 1a) is such that the mappings converge in the 5′–3′ direction, without any overlapping base mappings.

Paired-end overlap may indicate one of several potential problems with a paired-end mapping:

- The sequenced DNA fragment may have been shorter than the total length of the mates, so the mates’ sequences overlap.
- The mates may map within a larger repetitive region of the genome (e.g. within an extended tandem repeat) and the read aligner arbitrarily placed both mappings in the same location within the region.
- The inferred fragment length (TLEN) is no greater than the length of the longer mate (Fig. 1c); increased sequencer error rates have been associated with such very short fragment lengths (Tan *et al.* 2019).
- One or both mates are soft-clipped and the mate mappings are adjacent (or nearly so).

Overlapped mappings may impair a variant caller’s ability to use coverage to estimate variant (or variant allele) call probabilities. When a variant caller uses coverage to estimate variant-call probabilities at a particular location, it is in effect counting the number of DNA fragments that contain bases that differ from the reference genome. Since a pair of mates whose mappings overlap represent only one DNA fragment, pairs with overlapped mate mappings may skew the caller’s coverage estimates and lead to an increase in false positive variant calls.

Inaccuracies related to overlapping mate mappings can be addressed by excluding the more extreme forms of overlap from subsequent analysis. There are several ways to accomplish this:

- Configure read aligner constraints so that mate mappings with covering (Fig. 1c) or dovetailed (Fig. 1d) mapping topologies are not considered to be proper mappings.
- Set a minimum TLEN threshold to exclude paired-end mappings whose inferred fragment length is less than the minimum length of the mates being aligned.
- Filter SAM/BAM output to exclude mappings with TLEN below the minimum threshold.
- Configure the variant caller to ignore mappings with TLEN below the minimum threshold; if the variant caller uses a machine learning model, ensure that fragment length is incorporated into the model.
- Use a variant caller that uses heuristics to resolve base-call conflicts between overlapping mates (e.g. Illumina Corporation 2022a).

For example, Table 2 illustrates an increase in both recall and precision for variant identification in human chromosome 14 with the exclusion of proper mappings having TLEN less than the mate length. In this experiment, such mappings accounted for ∼1% of properly mapped pairs but over 15% of false positive variant calls.

**Table 2.** Variant-calling recall and precision (a) without constraints on TLEN and (b) excluding paired-end mappings having TLEN ≤ mate length.

	Pairs		Truth	TP	FP	Recall	Precision	F1	TiTv
(a) min TLEN = 1	342145691	INDEL	17445	17330	120	0.99341	0.99342	0.99342	
		SNP	108989	108497	643	0.99549	0.99411	0.99480	2.05704
(b) min TLEN = 152	338480432	INDEL	17445	17331	98	0.99346	0.99462	0.99404	
		SNP	108989	108504	544	0.99555	0.99501	0.99528	2.05819

*Notes:* Paired-end 151 bp reads from HG002 chromosome 14 aligned with BWA MEM to GRCh38. Variants from HaplotypeCaller using --min-fragment-length 152 to constrain TLEN.

### 2.3 Computational effort and variant-calling accuracy

Current sequencer technologies produce paired-end WGS reads with base miscall rates (error rates) of 0.5% or less, and short-read aligners commonly report high-similarity proper mappings with optimal or near-optimal alignment scores (AS) for over 90% of the reads in a WGS sequencer run (Mallick *et al.* 2016, Wilton *et al.* 2019). For this reason, aligners implement heuristics that favor the rapid discovery of mappings that contain few or no differences from the reference genome (Wilton and Szalay 2022). After reporting mappings for such reads, the aligner searches for mappings for the remaining subset of reads that contain indels or multiple base mismatches.

**Figure 2.**
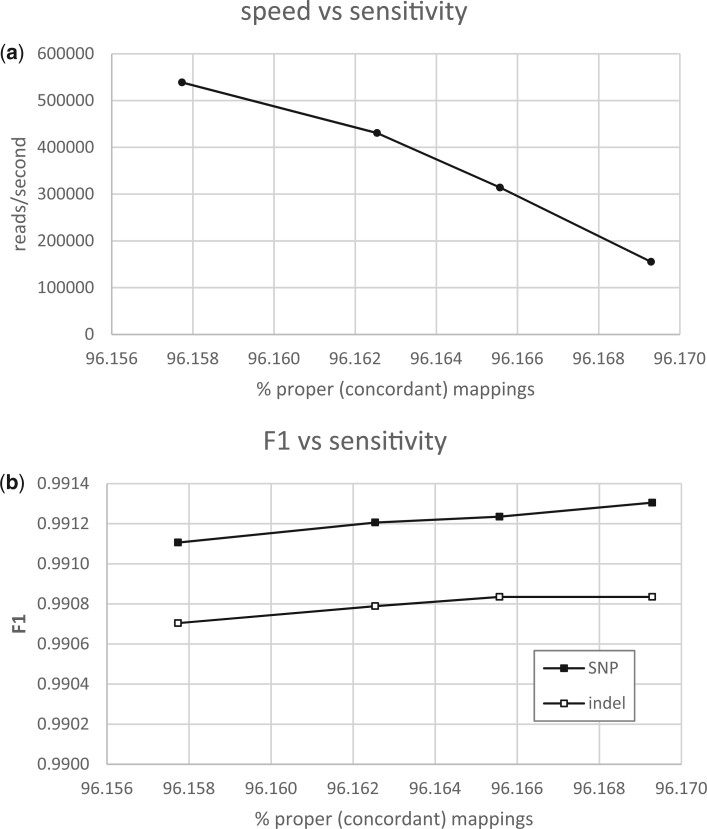
(a) Configuring a read aligner for increased sensitivity (computational effort) decreases aligner throughput and (b) produces a modest increase in variant-calling recall and precision. Reads from HG002 aligned to GRCh38 using Arioc with maxJ = {50, 100, 200, 1024} for gapped alignments. F1 (harmonic mean of recall and precision) computed with hap.py.

This subset of dissimilar—and thus hard-to-map—reads is important for variant discovery, but mapping them accounts for most of the computation time in short-read alignment. For this reason, aligner implementations expose runtime parameters that limit the computational effort involved and permit a performance tradeoff: a modest increase in recall and precision of variant identification requires significantly more alignment computation and lower aligner throughput (Fig. 2).

## 3 Alignment scoring and variant-calling accuracy

A variant caller initially identifies sites of potential variation by relying on the read aligner’s placement of read mappings in the reference genome. Read aligners use both a set of alignment scoring weights and a minimum AS threshold for reporting read mappings. These configurable parameters affect whether and where the aligner ultimately reports each read mapping. This affects a variant caller’s subsequent performance in terms of where it identifies sites of potential variation and how it characterizes variants at those sites.

### 3.1 Minimum AS

All general-purpose short-read aligners implement a runtime parameter that specifies a minimum AS threshold for reporting proper mappings. A lower AS threshold specifies that the aligner may report mappings for reads with lower similarity to the reference; a higher AS threshold requires that the aligner search for and report only mappings with higher similarity.

#### 3.1.1 The AS threshold facilitates aligner performance optimization

For “easy-to-map” read sequences with high similarity to the reference genome, the AS threshold does not matter because such reads have rapidly identifiable high-scoring mappings. But when a read sequence contains multiple differences from the reference genome, a lower AS threshold can increase speed by letting the aligner report lower-scoring mappings as soon as it discovers them. Conversely, with such reads, a higher AS threshold can improve mapping accuracy by causing the aligner to compute alignments at more candidate mapping locations before reporting the highest scoring mapping.

The AS threshold supports aligner performance optimization by controlling this tradeoff between computational effort and alignment accuracy. General-purpose short-read aligners are distributed with default or recommended AS threshold values that are fairly low so that the aligner can report mappings that contain a number of mismatches and/or gaps (Table 3). This produces very good (but submaximal) accuracy with high (but submaximal) throughput.

**Table 3.** Default AS threshold for three short-read aligners, for 150 bp read sequences.

	*W*_m_	*W*_x_	*W*_g_	*W*_s_	*V*_p_	*V*_t_	Mismatches	1-base gaps
Arioc	2	−6	−5	−3	300	150	18	15
Bowtie 2	2	−6	−5	−3	300	60	30	24
BWA-MEM	1	−4	−6	−1	150	30	24	15

*Note: W*_m_: match; *W*_x_: mismatch; *W*_g_: gap open; *W*_s_: gap space; *V*_p_: perfect AS; *V*_t_: AS threshold.

For variant-calling purposes, however, determining an optimal AS threshold poses a quandary. A higher AS threshold forces an aligner to search for and report higher-scoring mappings overall, so variant-calling accuracy is improved, yet interesting sequence variation may be found in hard-to-map read sequences that have lower ASs.

#### 3.1.2 A flexible AS threshold can improve variant-calling accuracy

Short-read aligners address this problem by implementing special-case AS threshold logic for paired-end mappings where only one mate is hard to map. Such mappings may be useful for identifying variants in variable or repetitive regions of the reference genome, so the aligner may report a proper paired-end mapping despite a subthreshold AS for the hard-to-map mate. For example, BWA-MEM reports “best” paired-end mappings using a heuristic that combines AS, a “maximal exact matches” score, and the expected DNA fragment length (Li 2013), while the AS threshold acts as a permissive, worst-case minimum. Arioc’s approach is to support an optional (lower) AS threshold for the hard-to-map mates in such pairs so that a higher AS threshold can still be used to tune overall mapping accuracy.

### 3.2 Alignment scoring weights

There is theoretical support for the idea that short-read mapping accuracy can be optimized with an appropriate choice of the scoring weights used for Smith–Waterman alignment (Vingron and Waterman 1994). In particular, one can alter the relative frequency with which an aligner reports SNPs and indels by using scoring weights with higher or lower penalties for mismatched bases (SNPs) relative to the penalty for gaps (insertions and deletions). In this way, an appropriate choice of scoring weights might improve variant-calling performance by causing the aligner’s base-by-base mappings to be consistent with the expected frequencies of SNPs and indels in the DNA samples being aligned.

However, high-accuracy variant callers do not identify SNPs and indels by using the aligner’s reported base-by-base mapping information. Instead, they use their own empirically optimized scoring weights to remap all the reads that span each potential variant site. This ensures consistent base-by-base mapping with repetitive read sequences and with complex variant genotypes.

#### 3.2.1 Scoring weights may affect mapping location

Alignment scoring weights can nevertheless affect variant-calling accuracy indirectly. Variant callers rely on the mapping location (RNAME, POS) reported by the aligner to identify reference-genome regions that may contain characterizable variants. When a read sequence has a high-scoring mapping in two similar (but not identical) reference genome regions, different alignment scoring weights can result in the aligner reporting a different highest scoring mapping location for the read, so variant calls at either of these alternative mapping locations may differ as well. Supplemental Fig. SF1 illustrates a case where different alignment scoring weights resulted in the presence or absence of a false positive variant identification.

#### 3.2.2 Scoring weights interact with the AS threshold

The configured minimum AS constrains the magnitude of individual scoring weights. For example, increasing the penalty for a mismatched base without lowering the AS threshold decreases the maximum number of mismatches that can be reported for a mapping. This can decrease the total number of proper mappings reported by the aligner for reads with multiple mismatches and borderline AS. Excluding these reads from subsequent analysis may lead to a decrease in false positive variant calls as the penalty increases (Supplementary Table ST5).

#### 3.2.3 When is scoring weight optimization worthwhile?

The default alignment scoring weights for a general-purpose short-read aligner are chosen empirically to maximize the sensitivity and specificity with which the aligner reports read-mapping locations (RNAME, POS). As Supplementary Table ST5 suggests, however, the default weights are not necessarily optimal for identifying both SNPs and indels, nor is the same set of scoring weights consistently optimal for all aligners. Nevertheless, since the effect of scoring weight optimization is limited primarily to a modest reduction of false positive variant calls, it is reasonable to expect “good enough” accuracy without attempting this optimization. But when variant discovery is directed toward a goal such as discovery of a specific variant type (e.g. SNPs), these benchmarking results indicate that scoring weight optimization can be beneficial.

## 4 MAPQ is essential but problematic

Short-read aligners report two distinct “quality” scores that variant callers rely upon in estimating variant-call error probabilities. BQS are computed during read sequencing and reported without modification by the aligner in the SAM/BAM QUAL field, which contains one BQS for each base in a read sequence. The BQS is an error probability: the sequencer’s estimate of the probability that a reported base call is wrong.

MAPQ is a short-read aligner’s estimate of the error probability associated with a read’s mapping location (RNAME, POS). An aligner computes MAPQ using a theoretical model that incorporates both a read’s highest scoring mapping and the ASs associated with its alternative lower-scoring mappings (Li *et al.* 2008, Frith 2020). The model also includes heuristics related to the number of alternative mappings, the number of reference genome bases covered by each mapping, and (for paired-end reads) the inferred fragment length (TLEN). The implementation of the model is empirically adjusted to produce a distribution of probability estimates that is consistent with the distribution of mapping locations observed with alignments of simulated reads derived from a reference genome.

### 4.1 MAPQ is a poor quantifier of probability

The heuristic nature of MAPQ computation implies an inherent uncertainty in the probability represented by MAPQ. MAPQ values are qualitatively consistent—that is, the mapping locations of high-MAPQ reads are more certainly correct than the mapping locations of low-MAPQ reads—but quantitatively unreliable as an error probability estimate (Wilton and Szalay 2022). Although it is possible to improve MAPQ accuracy through postprocessing (e.g. Ruffalo *et al.* 2012, Langmead 2017, Cline *et al.* 2020), this can require a significant amount of additional processing time.

These considerations make it hard to use MAPQ for its intended purpose as a quantitative read-placement error probability. Variant callers generally mitigate this problem by supporting configurable constraints on the usage of MAPQ values: a range of acceptable MAPQ values (i.e. to exclude reads with out-of-range MAPQ values from analysis), an option to use a read’s MAPQ as an upper limit on each of the BQS values in the read, or an option to exclude MAPQ from the statistical model used to compute variant quality scores.

### 4.2 Variant caller configuration constraints on MAPQ

General-purpose short-read aligners reliably report MAPQ values of 3 or less for reads that have high-scoring mappings at two or more different reference genome locations. This observation justifies excluding mappings with MAPQ ≤ 3 from any subsequent analysis. Additional gains in variant-calling accuracy may be obtained by using the distribution of MAPQ values actually reported by the aligner (e.g. Supplementary Script S11) to configure additional constraints on MAPQ in the variant caller.

This can be demonstrated using Bowtie 2 mappings analyzed with GATK HaplotypeCaller. Bowtie 2 reports MAPQ in the range 0–44, whereas HaplotypeCaller in its default configuration expects MAPQ values in the range 0–60. Variant-calling accuracy with this combination of tools is improved by linear scaling of the Bowtie 2 MAPQ values into the range 0–60 (Table 4a and b); a comparable increase in accuracy is obtainable by adjusting HaplotypeCaller’s runtime configuration (Table 4c).

**Table 4.** Configuration adjustments for expected MAPQ distribution.

	Truth	TP	FP	Recall	Precision	F1
(a) MAPQ 0–44						
INDEL	525469	517015	3981	0.98391	0.99266	0.98827
SNP	3365127	3302291	15031	0.98133	0.99547	0.98835
(b) MAPQ 0–60						
INDEL	525469	518172	4125	0.98611	0.99242	0.98926
SNP	3365127	3322286	15757	0.98727	0.99528	0.99126
(c) MAPQ 0–44, variant caller configured for MAPQ distribution						
INDEL	525469	518734	4491	0.98718	0.99176	0.98947
SNP	3365127	3329677	17185	0.98946	0.99487	0.99216

*Notes:* Reads from HG002, aligned with Bowtie 2, variants from GATK HaplotypeCaller. (a) MAPQ values 0–44, default HaplotypeCaller configuration. (b) MAPQ values linearly scaled to 0–60, default HaplotypeCaller configuration. (c) MAPQ values 0–44, HaplotypeCaller configured for MAPQ distribution and range 0–44 using –minimum-mapping-quality 14, --mapping-quality-threshold-for-genotyping 14, and –disable-cap-base-qualities-to-map-quality.

## 5 Choosing a reference genome

A reference genome is not a unique, immutable DNA sequence. Reference genomes may be revised to incorporate new information about individual diversity, to correct technical errors, and to contain information about common variation. In principle, such revisions should lead to improved performance of both read aligners and variant callers.

However, the choice of a reference genome involves considerations other than benchmark metrics of variant-calling accuracy. The existence of appropriate genome annotations may itself determine which reference genome must be used for variant analysis (Armstrong *et al.* 2019), regardless of the performance of the variant analysis toolchain. Also, corresponding subregions of different reference genome assemblies may differ in placement and in length (Salzberg 2019), so it is impossible to translate (“lift over”) genome locations (RNAME and POS) from one reference genome version to another while maintaining an identical set of variant annotations (Li *et al.* 2021).

These considerations may outweigh potential gains in the performance of variant-calling software. When the opportunity arises, however, an appropriate choice of reference genome may increase variant-calling accuracy.

### 5.1 GRCh38 versus GRCh37

In detailed comparisons of the two most recent major releases of the human reference genome, GRCh37 (NCBI 2023a) and GRCh38 (NCBI 2023b), the use of GRCh38 improves both recall and precision in variant discovery when compared with GRCh37 (Schneider *et al.* 2017). This improvement may be due to the elimination of errors in the reference genome assembly or to more accurate base-by-base representations of paralogous and highly variable regions of the reference genome, or it may simply be attributable to the observation that GRCh38 is more complete (Li 2014a) in that it covers 2.3% more of the reference genome sequence than does GRCh37.

In our analysis toolchain, all three aligners reported an increased number of proper mappings with GRCh38 (Supplementary Table ST6) as well as an increase in the fraction of reads having perfect or near-perfect proper mappings (Supplementary Table ST7). Variant-calling precision also increased using GRCh38 with all three aligners (Table 5), presumably because a larger fraction of reads map correctly with GRCh38, thereby decreasing the number of false positive variant calls.

**Table 5.** Variant-calling accuracy (precision and *F*-measure) is greater with GRCh38 than with GRCh37.

		Recall		Precision		F1	
		GRCh37	GRCh38	GRCh37	GRCh38	GRCh37	GRCh38
Arioc	INDEL	0.99216	0.99183	0.98756	0.98984	0.98985	0.99084
	SNP	0.99003	0.98998	0.99072	0.99263	0.99038	0.99130
BWA-MEM	INDEL	0.99178	0.99143	0.98471	0.98979	0.98824	0.99061
	SNP	0.98958	0.98996	0.98799	0.99202	0.98878	0.99099
Bowtie 2	INDEL	0.98753	0.98718	0.99108	0.99176	0.98930	0.98947
	SNP	0.99020	0.98946	0.99328	0.99487	0.99174	0.99216

*Notes*: Reads from HG002 mapped with Arioc, BWA-MEM, and Bowtie 2. Variants called with HaplotypeCaller.

### 5.2 GRCh38 versus T2T-CHM13

The release of the complete “telomere-to-telomere” human reference genome (Nurk *et al.* 2022, NCBI 2023c) was accompanied by evidence that its use leads to additional improvements in variant discovery and in the accuracy of variant characterization (Aganezov *et al.* 2022) in comparison with GRCh38. Although genome-wide benchmark variant annotations for T2T-CHM13 are not yet available, the addition of nearly 6% of additional sequence to the reference genome is expected to permit read aligners to report an increased number of proper mappings (Supplementary Tables ST6 and ST7), with consequent improvements in variant-calling accuracy.

### 5.3 Nonlinear reference genomes

As experience with GRCh38 and T2T-CHM13 demonstrates, a read aligner’s ability to discover high-scoring mappings is facilitated by reference genomes that cover as much genome sequence as possible without errors in base sequence or in assembly. In principle, further gains in alignment sensitivity ought to be achievable with a reference genome that more fully represents normal, nonpathological variation—that is, with a reference genome that contains alternative sequence for paralogous and highly variable reference genome regions (Jäger *et al.* 2016) and for areas of the genome susceptible to significant structural variation (Feuk *et al.* 2006).

But alternative reference sequence impedes the recognition of short variants because it complicates read alignment. Algorithms for identifying locations at which to compute alignments (Altschul *et al.* 1990) and computing base-by-base mappings (Smith and Waterman, 1981) require significant modification or postprocessing to be useful with nonlinear reference sequence. Disambiguating mappings to alternative reference sequences can add significant complexity to a variant analysis workflow (e.g. Broad Institute 2022, Illumina Corporation 2022b). Paired-end short-read alignment is problematic whenever the mates of a paired-end read map to different alternative reference sequences or when mate mappings straddle a region that contains alternative sequences of different lengths; long-read sequencing might be better suited to providing accurate coverage of such regions (Olson *et al.* 2023).

Alternative reference sequence also complicates an aligner’s computation of MAPQ. Because alternative sequences in a given region are frequently similar to each other, a read aligner can discover plausible high-scoring mappings for the same read in two or more alternative sequences. In this situation, an aligner implementation must determine whether to report one or multiple mappings, and compute MAPQ accordingly (Li 2014b).

#### 5.3.1 Alt contigs

Without this additional logic, the aligner can report reads as having multiple, high-scoring mappings at independent reference genome locations, with a correspondingly low MAPQ; a variant caller will subsequently exclude such low-MAPQ mappings from subsequent analysis. The resulting decrease in variant-calling sensitivity (recall) is non-negligible, even though alt contig sequence may cover only a small fraction of the reference genome (e.g. about 3% with GRCh38). This has been observed with whole exome sequencing reads (Jia *et al.* 2020) and can be seen in our WGS analysis pipeline (Table 6).

**Table 6.** Variant-calling accuracy with reads aligned using alt contigs without postprocessing mapping locations and MAPQ.

	Truth	TP		FP		Recall		Precision		F1 (*F*-measure)	
		GRCh38	GRCh38+alt	GRCh38	GRCh38+alt	GRCh38	GRCh38+alt	GRCh38	GRCh38+alt	GRCh38	GRCh38+alt
INDEL	17445	17334	16954	90	87	0.99364	0.97185	0.99506	0.99512	0.99435	0.98335
SNP	108989	108397	105632	226	230	0.99457	0.96920	0.99792	0.99783	0.99624	0.98330

*Notes*: Reads from HG002 mapped to GRCh38 (chr14 only), with and without ALT_REF_LOCI reference sequences. Variants called with HaplotypeCaller.

#### 5.3.2 Graph-based genomes

These problems may be addressed through the use of a graph-based reference genome (e.g. Liao *et al.* 2023) with a read aligner that incorporates heuristics that search for mappings only in plausible subsets of the alternative sequence in the genome (Sirén *et al.* 2021). This technology is not yet mature. Its eventual applicability will depend on the development of practical techniques for recognizing and cataloging variation in regions of alternative sequence, particularly where alternative sequence representations in a reference genome region are so similar that reads from a single WGS sample may map to each alternative.

## 6 Performance optimization

Software performance optimization customarily focuses on resource utilization: memory usage, data storage space, CPU or GPU computation, and so on. When evaluating short-read aligner performance, the most useful metrics for optimization emphasize the tradeoff between resource utilization (e.g. speed) and software functionality (e.g. sensitivity). In the context of variant analysis, however, performance evaluation customarily prioritizes variant-calling accuracy, and performance tuning focuses on configuring the read aligner to produce output that the variant caller is configured to use as input.

### 6.1 Are default parameter settings optimal?

Read aligners and variant callers are distributed with default runtime configuration settings that usually produce acceptable results even without optimization. A number of studies have evaluated the performance of variant analysis toolchains using short-read aligners as “black box” tools, without consideration of the effect of aligner runtime configuration and performance on downstream variant analysis (e.g. Chen *et al.* 2019, Hwang *et al.* 2019, Schilbert *et al.* 2020, Zhao *et al.* 2020, Zanti *et al.* 2021, Barbitoff *et al.* 2022, Betschart *et al.* 2022). The inconsistent results among such studies imply that default parameter settings are not always optimal in every variant analysis toolchain. In practice, it is important to use variant-calling results to validate both aligner and variant caller performance, not only to optimize results but also to avoid configuration errors that compromise performance.

### 6.2 Are some software tools more performant than others?

In the past decade, dozens of general-purpose read aligner implementations have been developed, but only a handful are actively supported by their developers, take full advantage of computing hardware resources, produce acceptable results for hard-to-compute input (i.e. mappings for difficult-to-align read sequences), generate all of the metadata required for high-accuracy variant calling, and provide a useful variety of runtime configuration parameters. Similar desiderata apply to variant callers, although there exists a wide variety of specialized implementations (e.g. for germline variants, exome-only variants, structural variants, etc.).

**Figure 3.**
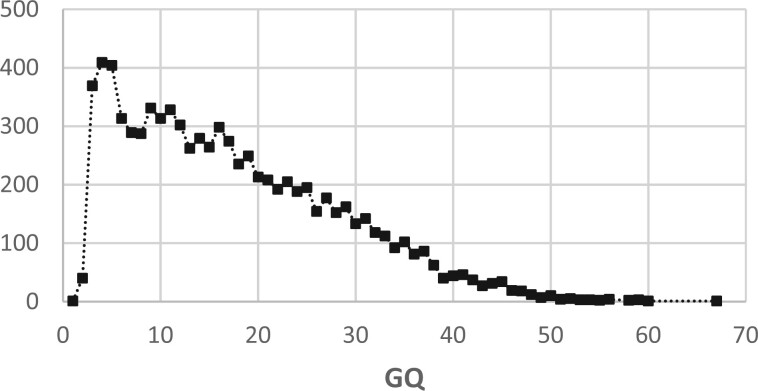
Frequency distribution of GQ values for TP variants reported by DeepVariant and not reported by HaplotypeCaller. Reads from HG002 aligned with Arioc to GRCh38

Software tools that meet these criteria can generate accurate variant-calling results (Olson *et al.* 2022). The differences among such tools are found mainly in the results they produce with hard-to-align input (read mappings with multiple mismatches or indels; reads mapped to repetitive regions of the reference genome), which leads to variant calls associated with low MAPQ scores (due to low coverage or to low-quality read mappings). For example, of the three variant callers we evaluated, DeepVariant consistently reported results with more true positive and fewer false positive variant calls than the other two variant callers, although the additional TP variants discovered only by DeepVariant were predominantly associated with low-quality scores (Fig. 3).

### 6.3 How to optimize read aligner performance

In a variant analysis toolchain, maximum accuracy in variant calling is obtainable by configuring both the read aligner and the variant caller to be consistent in their handling of read-mapping metadata (MAPQ, TLEN, minimum AS). The optimal configuration of each of these tools always depends on the particulars of paired-end mapping topology, alignment scoring, MAPQ computation, and a specific reference genome.

Tuning these configurations is trial-and-error work, but it is not time-consuming when it is done using only a small subset of the sequencer reads. Read aligners and variant callers both provide a variety of runtime configuration parameters that control the production of these metadata by the read aligner and their usage by the variant caller, but only a small subset of these parameters (Supplementary Tables ST2 and ST3) is essential to ensuring that read-aligner output becomes optimal variant-caller input.

## Supplementary Material

btad480_Supplementary_Data

## Data Availability

All data are incorporated into the article and its online supplementary material.
