# Supplementary material for: Short-read aligner performance in germline variant identification
Source: Bioinformatics. 2023 Aug 1;39(8):btad480. doi: 10.1093/bioinformatics/btad480 (PMC10421969; doi:10.1093/bioinformatics/btad480)
Supplement: btad480_Supplementary_Data [file btad480_supplementary_data.pdf]

Supplementary Information

Short-read aligner performance in germline variant identification

Richard Wilton and Alexander S. Szalay

**Linux bash script examples**	
	Open-source software license
Script S1	Short-read alignment with Arioc
Script S2	Short-read alignment with Bowtie 2
Script S3	Short-read alignment with BWA-MEM
Script S4	Variant calling with GATK HaplotypeCaller
Script S5	Variant calling with DeepVariant
Script S6	Variant calling with FreeBayes
Script S7	Benchmarking with hap.py
Script S8	Computing variant hard-filtering parameters with vcfeval
Script S9	Filtering variants with GATK VariantFiltration
Script S10	AS frequency distribution
Script S11	MAPQ frequency distribution
Script S12	Variant annotation frequency distribution
Script S13	Differential GQ frequency distribution
**Tables**	
Table T1	Software versions and download URLs
Table T2	Important runtime configuration parameters for general-purpose short-read aligners
Table T3	Important alignment-related runtime configuration parameters for germline variant callers
Table T4	Effect of increased MAPQ threshold on variant calling accuracy
Table T5	Effect of alignment scoring weights on alignment sensitivity and variant calling accuracy
Table T6	Reference genome: fraction of reads with proper mappings
Table T7	Reference genome: fraction of reads with perfect or near-perfect mappings
**Figures**	
Figure F1	Alignment scoring weights can affect read mapping location
Figure F2	GQ increases with higher MAPQ

Open-source software license

The BSD 3-Clause License applies to the source-code examples labeled Script S10, Script S11, and Script S12.

Copyright notice: the software examples in this Supplemental Material are copyright (c) 2023 Johns Hopkins University.

Redistribution and use in source and binary forms, with or without modification, are permitted provided that the following conditions are met:

1. Redistributions of source code must retain the above copyright notice, this list of conditions and the following disclaimer.

2. Redistributions in binary form must reproduce the above copyright notice, this list of conditions and the following disclaimer in the documentation and/or other materials provided with the distribution.

3. Neither the name of the copyright holder nor the names of its contributors may be used to endorse or promote products derived from this software without specific prior written permission.

THIS SOFTWARE IS PROVIDED BY THE COPYRIGHT HOLDERS AND CONTRIBUTORS "AS IS" AND ANY EXPRESS OR IMPLIED WARRANTIES, INCLUDING, BUT NOT LIMITED TO, THE IMPLIED WARRANTIES OF MERCHANTABILITY AND FITNESS FOR A PARTICULAR PURPOSE ARE DISCLAIMED. IN NO EVENT SHALL THE COPYRIGHT HOLDER OR CONTRIBUTORS BE LIABLE FOR ANY DIRECT, INDIRECT, INCIDENTAL, SPECIAL, EXEMPLARY, OR CONSEQUENTIAL DAMAGES (INCLUDING, BUT NOT LIMITED TO, PROCUREMENT OF SUBSTITUTE GOODS OR SERVICES; LOSS OF USE, DATA, OR PROFITS; OR BUSINESS INTERRUPTION) HOWEVER CAUSED AND ON ANY THEORY OF LIABILITY, WHETHER IN CONTRACT, STRICT LIABILITY, OR TORT (INCLUDING NEGLIGENCE OR OTHERWISE) ARISING IN ANY WAY OUT OF THE USE OF THIS SOFTWARE, EVEN IF ADVISED OF THE POSSIBILITY OF SUCH DAMAGE.

```xml
<?xml version="1.0" encoding="utf-8"?>

<AriocP gpuMask="0x000F" batchSize="48K" verboseMask="0xE0004007">

  <R>/path/to/encoded/reference/genome</R>

  <nongapped seed="ssi84_2_30" maxJ="*" maxMismatches="2" />

  <gapped seed="hsi20_0_30"
          Wmxgs="2_8_5_3"
          Vt="L,0,1.5" Vtw="L,0,0.3"
          maxJ="1024" seedDepth="6" AtO="2" />

  <Q filePath="/path/to/encoded/reads" errorRate="0.7">
    <paired srcId="1" subId="1" srcInfo="pFDA:HG002">
      <file>HG002.novaseq.pcr-free.35x.R1</file>
      <file>HG002.novaseq.pcr-free.35x.R2</file>
    </paired>
  </Q>

  <A baseName="HG002" basePath="/path/to/output/directory" overwrite="true"
     pairOrientation="c" pairCollision="o" pairFragmentLength="0-1500"
     maxAperRead="2">
    <sam report="c">output_directory_name</sam>
    <sam report="d">output_directory_name</sam>
    <sam report="r">output_directory_name</sam>
    <sam report="u">output_directory_name</sam>
  </A>

</AriocP>
```

Script S1. Short-read alignment with Arioc. (Placeholders replace actual file paths.)

```
#
# doAlign.sh
#

BTHOME=/path/to/bowtie2-2.4.5-linux-x86_64
RDIR=/path/to/encoded/reference/genome
QDIR=/path/to/reads
ADIR=/path/to/output/directory

echo "Start on $HOSTNAME at $(date +%FT%T)" > doAlign.log

$BTHOME/bowtie2 \
 --ignore-quals \
 --local \
 --ma 2 \
 --mp 6 \
 --rdg 13,3 \
 --rfg 13,3 \
 --np 6 \
 --score-min L,0,1.5 \
 --gbar 1 \
 --minins 0 \
 --maxins 1500 \
 --fr \
 --time \
 --threads 64 \
 --xeq \
 --rg-id rg1 \
 --rg SM:HG002 \
 -x $RDIR \
 -1 $QDIR/HG002.novaseq.pcr-free.35x.R1.fastq \
 -2 $QDIR/HG002.novaseq.pcr-free.35x.R2.fastq \
 -S $ADIR/HG002.sam \
 &>> doAlign.log

echo "Done at $(date +%FT%T)" &>> doAlign.log
```

Script S2. Short-read alignment with Bowtie 2. (Placeholders replace actual file paths.)

```
#
# doAlign.sh
#

BWAEXE=/path/to/bwa-mem2-2.2.1_x64-linux/bwa-mem2
IDXBASE=/path/to/encoded/reference/genome
QBASE=/path/to/reads
ABASE=/path/to/output/directory

# align
$BWAEXE mem \
 -t 64 \
 -R '@RG\tID:rg1\tSM:HG002' \
 -v 3 \
 -M \
 -A 1 \
 -B 12 \
 -O 6 \
 -E 1 \
 $IDXBASE \
 -o $ABASE/HG002.sam \
 $QBASE/HG002.novaseq.pcr-free.35x.R1.fastq \
 $QBASE/HG002.novaseq.pcr-free.35x.R2.fastq \
 &> doAlign.log

echo "Done at $(date +%FT%T)" &>> doAlign.log
```

Script S3. Short-read alignment with BWA-MEM2. (Placeholders replace actual file paths.)

```
#
# doCaller.sh
#

GATKROOT=/path/to/gatk-4.3.0.0
BAM=/path/to/HG002.cfus.bam
R=/path/to/GRCh38.p14.fna
intervalsList='-L chr1 -L chr2 -L chr3 -L chr4 -L chr5 -L chr6 -L chr7 -L chr8 -L chr9 -L chr10 -L chr11 -
L chr12 -L chr13 -L chr14 -L chr15 -L chr16 -L chr17 -L chr18 -L chr19 -L chr20 -L chr21 -L chr22'
OUTDIR=vcf1

# output filenames
GVCF="HG002.g.vcf.gz"
VCF="HG002.vcf.gz"

echo "Start at $(date +%FT%T)" > doCaller.log

# call variants with GATK HaplotypeCaller
echo 'Calling variants with HaplotypeCaller...'
$GATKROOT/gatk HaplotypeCaller \
  --reference ${R} \
  ${intervalsList} \
  --emit-ref-confidence GVCF \
  --sample-name HG002 \
  --smith-waterman FASTEST_AVAILABLE \
  --native-pair-hmm-threads 56 \
  --read-filter FragmentLengthReadFilter --min-fragment-length 152 \
  --minimum-mapping-quality 20 \
  --mapping-quality-threshold-for-genotyping 20 \
  --input $BAM \
  --output $OUTDIR/$GVCF \
  &>> doCaller.log

rval=$?
echo "HaplotypeCaller ends (${rval})" >> doCaller.log

if [ ${rval} != 0 ]
then
    exit ${rval}
fi

echo '' >> doCaller.log
echo 'Running GATK GenotypeGVCFs...' >> doCaller.log

$GATKROOT/gatk GenotypeGVCFs \
 --reference ${R} \
 --variant $OUTDIR/$GVCF \
 --output $OUTDIR/$VCF \
  &>> doCaller.log

echo >> doCaller.log
echo 'Unzip VCF output...' >> doCaller.log
gunzip -f -k $OUTDIR/$VCF

echo "Done at $(date +%FT%T)" >> doCaller.log
```

Script S4. Variant calling with GATK HaplotypeCaller. (Placeholders replace actual file paths.)

```
#
# doCaller.sh
#

BIN_VERSION=1.4.0
N_SHARDS=56
REGIONS='chr1 chr2 chr3 chr4 chr5 chr6 chr7 chr8 chr9 chr10 chr11 chr12 chr13 chr14 chr15 chr16 chr17
chr18 chr19 chr20 chr21 chr22'

# host filesystem paths
HFS_INPUT=/path/to/mapped/reads
HFS_OUTPUT=${PWD}/vcf1
HFS_LOG=${PWD}
HFS_REFERENCE=/path/to/reference/genome

# docker filesystem paths
DFS_INPUT=/input
DFS_OUTPUT=/output
DFS_REFERENCE=/reference

# input filenames
REFERENCE_GENOME=GRCh38.p14.fna
MAPPINGS=HG002.cfus.bam

# output filenames
VCF=HG002.vcf.gz

echo "Start on $HOSTNAME at $(date +%FT%T)"

# ensure that the output directory exists
mkdir -v -p -m=777 ${HFS_OUTPUT}

# call variants with DeepVariant
docker run --gpus 1 \
  -v "${HFS_INPUT}":"${DFS_INPUT}" \
  -v "${HFS_OUTPUT}":"${DFS_OUTPUT}" \
  -v "${HFS_REFERENCE}":"${DFS_REFERENCE}" \
  -v "${PWD}/tmp":"/tmp" \
  google/deepvariant:"${BIN_VERSION}-gpu" \
  /opt/deepvariant/bin/run_deepvariant \
  --model_type WGS \
  --ref "${DFS_REFERENCE}/${REFERENCE_GENOME}" \
  --reads "${DFS_INPUT}/${MAPPINGS}" \
  --regions "${REGIONS}" \
  --output_vcf "${DFS_OUTPUT}/${VCF}" \
  --num_shards=${N_SHARDS} \
  2>&1 | tee "${HFS_LOG}/doCaller.log"

rval=$?
echo "DeepVariant ends (${rval})" >> doCaller.log

if [ ${rval} != 0 ]
then
    exit ${rval}
fi

echo '' >> doCaller.log
echo 'Unzip VCF output...' >> doCaller.log
gunzip -f -k ${HFS_OUTPUT}/${VCF}

echo "Done at $(date +%FT%T)"
```

Script S5. Variant calling with DeepVariant. (Placeholders replace actual file paths.)

```
#
# doCaller.parallel.sh
#
# Notes:
#  The recommended manner of splitting things up is by fixed-size chunks (e.g. 500,000 bases) per
#   concurrent instance of freebayes, e.g.,
#
#   $FBDIR/freebayes-parallel <($FBDIR/fasta_generate_regions.py ${REFERENCE}.fai 500000) ${CONCURRENCY}
#    -f ${REFERENCE} ${MAPPINGS} > ${VCF}
#
#  Here we settle for some inefficiency by using chromosomes as chunks, which is far from optimal but
#   good enough for our purposes.
#
#  The freebayes-parallel python scripts require python 3.
#

FBDIR=/path/to/freebayes-1.3.6

REFERENCE=/path/to/GRCh38.p14.fna
MAPPINGS=/path/to/HG002.cfus.bam
OUTDIR=vcf1
WGSSAMPLE=HG002
VCF=${OUTDIR}/${WGSSAMPLE}.vcf

CONCURRENCY=22

# freebayes default minimum MAPQ is 1
MINMAPQ=1

# freebayes default minimum BQS is 0
MINBQS=0

echo "Start at $(date +%FT%T)" > doCaller.log
echo '' >> doCaller.log

# echo freebayes version
VER=$(~/tools/freebayes-1.3.6/freebayes --version | grep -o -E 'v([0-9\.])+')
echo "Using freebayes ${VER} with the following arguments:" >> doCaller.log

ARGS="--fasta-reference $REFERENCE \
      --bam $MAPPINGS \
      --min-mapping-quality ${MINMAPQ} \
      --use-mapping-quality \
      --genotype-qualities \
      --strict-vcf"
echo ${ARGS} >> doCaller.log

# use freebayes to call variants and generate a VCF
# (The file chr1_22.dat is a newline-terminated list of chromosome names chr1-chr22.)

echo "Using freebayes-parallel for ${CONCURRENCY} concurrent instances..." >> doCaller.log
$FBDIR/freebayes-parallel <(cat $FBDIR/chr1_22.dat) ${CONCURRENCY} ${ARGS} > ${VCF}

echo '' >> doCaller.log
echo "Done at $(date +%FT%T)" >> doCaller.log
```

Script S6. Variant calling with FreeBayes. (Placeholders replace actual file paths.)

```
#
# doEval1.sh
#
#  Benchmark VCF and BED files downloaded from ftp://trace.ncbi.nlm.nih.gov:
#   /ReferenceSamples/giab/release/AshkenazimTrio/HG002_NA24385_son/NISTv4.2.1/GRCh38

RTGEXE=/path/to/rtg-tools-3.12.1/rtg
SDFGENOME=/path/to/RTG/R/GRCh38/GRCh38.p14.sdf
REFERENCE=/path/to/GATK/GRCh38/GRCh38.p14.fna
TRUTH=/path/to/HG002_GRCh38_1_22_v4.2.1_benchmark.vcf.gz
FP=/path/to/HG002/HG002_GRCh38_1_22_v4.2.1_benchmark_noinconsistent.bed

VCFROOT=${PWD}/vcf1
VCFSTUB=HG002
QUERY=$VCFROOT/$VCFSTUB.vcf
LOCATIONS=chr1,chr2,chr3,chr4,chr5,chr6,chr7,chr8,chr9,chr10,chr11,chr12,chr13,chr14,chr15,chr16,chr17,chr
18,chr19,chr20,chr21,chr22

GATKROOT=/path/to/gatk-4.3.0.0
SNPS=$VCFROOT/$VCFSTUB.snps.vcf
INDELS=$VCFROOT/$VCFSTUB.indels.vcf
OUTDIR=eval1

# evaluate raw variant calls
hap.py \
 --reference $REFERENCE \
 --location $LOCATIONS \
 --false-positives $FP \
 --report-prefix $OUTDIR/${VCFSTUB} \
 --engine vcfeval \
 --engine-vcfeval-path $RTGEXE \
 --engine-vcfeval-template $SDFGENOME \
 $TRUTH \
 $QUERY \
 &>> doEval1.log

# split SNPs and INDELs
$GATKROOT/gatk SelectVariants \
  --variant $QUERY \
  --select-type-to-include SNP \
  --output $SNPS \
 &>> doEval1.log

$GATKROOT/gatk SelectVariants \
  --variant $QUERY \
  --select-type-to-include INDEL \
  --select-type-to-include MIXED \
  --output $INDELS \
 &>> doEval1.log

# RTG wants bgzip'd VCF files
$RTGEXE bgzip -c $SNPS > $SNPS.gz
$RTGEXE index $SNPS.gz &>> doEval1.log
$RTGEXE bgzip -c $INDELS > $INDELS.gz
$RTGEXE index $INDELS.gz &>> doEval1.log
```

Script S7. Benchmarking with hap.py. (Placeholders replace actual file paths.)

```
#
# doRTG.sh
#

SDFGENOME=/path/to/GRCh38.p14.sdf
BEDREGIONS=/path/to/HG002_GRCh38_1_22_v4.2.1_benchmark_noinconsistent.bed
VCFGOLD=/path/to/HG002_GRCh38_1_22_v4.2.1_benchmark.vcf.gz
RTGEXE=/path/to/rtg-tools-3.12.1/rtg

VCFSTUB=vcf1/HG002
PATHOUTSTUB=evalRTG/HG002

# SNPs
echo 'Estimating hard-filter cutpoints for SNPs...'
$RTGEXE version | grep -A 4 'Product:' &> doRTG.s.log
echo '' >> doRTG.s.log

### echo '***** SNP *****' >> doRTG.s.log
VCFCALLS=$VCFSTUB.snps.vcf.gz
PATHOUT=${PATHOUTSTUB}/s
rm -f -r $PATHOUT

echo 'INFO.QD' >> doRTG.s.log
$RTGEXE vcfeval --vcf-score-field INFO.QD --output $PATHOUT/INFO.QD \
 --template $SDFGENOME --baseline $VCFGOLD --evaluation-regions $BEDREGIONS --calls $VCFCALLS \
 &>> doRTG.s.log

echo 'QUAL' >> doRTG.s.log
$RTGEXE vcfeval --vcf-score-field QUAL --output $PATHOUT/QUAL \
 --template $SDFGENOME --baseline $VCFGOLD --evaluation-regions $BEDREGIONS --calls $VCFCALLS \
 &>> doRTG.s.log

echo 'INFO.MQ' >> doRTG.s.log
$RTGEXE vcfeval --vcf-score-field INFO.MQ --output $PATHOUT/INFO.MQ \
 --template $SDFGENOME --baseline $VCFGOLD --evaluation-regions $BEDREGIONS --calls $VCFCALLS \
 &>> doRTG.s.log

# INDELs
echo 'Estimating hard-filter cutpoints for INDELs...'
$RTGEXE version | grep -A 4 'Product:' &> doRTG.i.log
echo '' >> doRTG.i.log

echo '***** INDEL *****' >> doRTG.i.log
VCFCALLS=$VCFSTUB.indels.vcf.gz
PATHOUT=${PATHOUTSTUB}/i
rm -f -r $PATHOUT

echo 'INFO.QD' >> doRTG.i.log
$RTGEXE vcfeval --vcf-score-field INFO.QD --output $PATHOUT/INFO.QD \
 --template $SDFGENOME --baseline $VCFGOLD --evaluation-regions $BEDREGIONS --calls $VCFCALLS \
 &>> doRTG.i.log

echo 'QUAL' >> doRTG.i.log
$RTGEXE vcfeval --vcf-score-field QUAL --output $PATHOUT/QUAL \
 --template $SDFGENOME --baseline $VCFGOLD --evaluation-regions $BEDREGIONS --calls $VCFCALLS \
 &>> doRTG.i.log

echo 'INFO.MQ' >> doRTG.i.log
$RTGEXE vcfeval --vcf-score-field INFO.MQ --output $PATHOUT/INFO.MQ \
 --template $SDFGENOME --baseline $VCFGOLD --evaluation-regions $BEDREGIONS --calls $VCFCALLS \
 &>> doRTG.i.log

# scrape the output logs
echo 'Generating doVcfFilter.sh...'
x1='Variants in this region will not be included in results'
x2='problematic baseline variant'
x3='not thresholded in ROC data files'
```

```
INFO_QDi=$(grep -v "${x1}" doRTG.i.log | grep -v "${x2}" | grep -v "${x3}" | grep -A 4 'INFO.QD' | \
 tail -1 | grep -E -o [0-9\.]+ | head -1)
QUALi=$(grep -v "${x1}" doRTG.i.log | grep -v "${x2}" | grep -v "${x3}" | grep -A 4 'QUAL' | \
 tail -1 | grep -E -o [0-9\.]+ | head -1)
INFO_MQi=$(grep -v "${x1}" doRTG.i.log | grep -v "${x2}" | grep -v "${x3}" | grep -A 4 'INFO.MQ' | \
 tail -1 | grep -E -o [0-9\.]+ | head -1)

INFO_QDs=$(grep -v "${x1}" doRTG.s.log | grep -v "${x2}" | grep -v "${x3}" | grep -A 4 'INFO.QD' | \
 tail -1 | grep -E -o [0-9\.]+ | head -1)
QUALs=$(grep -v "${x1}" doRTG.s.log | grep -v "${x2}" | grep -v "${x3}" | grep -A 4 'QUAL' | \
 tail -1 | grep -E -o [0-9\.]+ | head -1)
INFO_MQs=$(grep -v "${x1}" doRTG.s.log | grep -v "${x2}" | grep -v "${x3}" | grep -A 4 'INFO.MQ' | \
 tail -1 | grep -E -o [0-9\.]+ | head -1)

echo " INDELs: INFO.QD ${INFO_QDi}, QUAL ${QUALi}, INFO.MQ ${INFO_MQi}"
echo " SNPs  : INFO.QD ${INFO_QDs}, QUAL ${QUALs}, INFO.MQ ${INFO_MQs}"

# do replacements to build a bash script for filtering the VCF results
sed 's/$(maxJ)/'${maxJ}'/g' doVcfFilter.template.sh \
 | sed 's/doVcfFilter.template.sh/doVcfFilter.sh (generated from template)/g' \
 | sed 's/$(INFO.QD.i)/'${INFO_QDi}'/g' \
 | sed 's/$(QUAL.i)/'${QUALi}'/g' \
 | sed 's/$(INFO.MQ.i)/'${INFO_MQi}'/g' \
 | sed 's/$(INFO.FS.i)/'${INFO_FSi}'/g' \
 | sed 's/$(INFO.QD.s)/'${INFO_QDs}'/g' \
 | sed 's/$(QUAL.s)/'${QUALs}'/g' \
 | sed 's/$(INFO.MQ.s)/'${INFO_MQs}'/g' \
 | sed 's/$(INFO.FS.s)/'${INFO_FSs}'/g' \
 > doVcfFilter.sh
```

Script S8. Computing variant hard-filtering parameters with vcfeval. This script processes a template (Script S9) to generate an executable bash script. (Placeholders replace actual file paths.)

```
#
# doVcfFilter.template.sh
#

GATKROOT=/path/to/gatk-4.3.0.0

VCF1ROOT=/path/to/vcf1
VCF2ROOT=/path/to/vcf2
VCFSTUB=HG002

# filter SNPS
$GATKROOT/gatk VariantFiltration \
 --variant $VCF1ROOT/$VCFSTUB.snps.vcf \
 --filter-name 'QD'     --filter-expression 'INFO.QD < $(INFO.QD.s)' \
 --filter-name 'QUAL'   --filter-expression 'QUAL < $(QUAL.s)' \
 --filter-name 'MQ'     --filter-expression 'INFO.MQ < $(INFO.MQ.s)' \
 --output $VCF2ROOT/$VCFSTUB.snps.f.vcf.gz \
 &> doVcfFilter.log

# filter INDELS
$GATKROOT/gatk VariantFiltration \
 --variant $VCF1ROOT/$VCFSTUB.indels.vcf \
 --filter-name 'QD'     --filter-expression 'INFO.QD < $(INFO.QD.i)' \
 --filter-name 'QUAL'   --filter-expression 'QUAL < $(QUAL.i)' \
 --filter-name 'MQ'     --filter-expression 'INFO.MQ < $(INFO.MQ.i)' \
 --output $VCF2ROOT/$VCFSTUB.indels.f.vcf.gz \
 &>> doVcfFilter.log

# merge into a single VCF
$GATKROOT/gatk MergeVcfs \
 --INPUT $VCF2ROOT/$VCFSTUB.snps.f.vcf.gz \
 --INPUT $VCF2ROOT/$VCFSTUB.indels.f.vcf.gz \
 --OUTPUT $VCF2ROOT/$VCFSTUB.f.vcf.gz \
 &>> doVcfFilter.log
```

Script S9. Filtering variants with GATK VariantFiltration. This template is processed by Script S8 to produce an executable bash script. (Placeholders replace actual file paths.)

```
#
# asd.awk
#

BEGIN {
    stderr = "/dev/stderr";
}

{
    # ignore SAM headers
    if( substr( $0, 1, 1 ) == "@" )
        next;

    # isolate interesting fields
    if( match( $0, /AS:i:([0-9]+)/, aAS ) )
    {
        # accumulate AS counts
        AS = aAS[1];
        nAS[AS]++ ;

        # count SAM records
        nSamRecords++ ;
    }
}

END {
    printf( "Total lines processed: %d\n", NR ) > stderr;
    printf( "Total SAM records: %d\n", nSamRecords ) > stderr;

    printf( "----\n" ) > stderr;
    printf( "AS\tn\n" ) > stderr;

    # find minimum and maximum AS
    ASmin = 9999;
    ASmax = 0;
    for( AS in nAS )
    {
        if( int(AS) < int(ASmin) )
            ASmin = AS;
        if( int(AS) > int(ASmax) )
            ASmax = AS;
    }

    for( AS=int(ASmin); AS<=int(ASmax); AS++ )
        printf( "%4d\t%d\n", AS, nAS[AS] ) > stderr;

    printf( "Done.\n" ) > stderr;
}
```

Script S10. Distribution of AS values in a SAM file.

```
#
# mqd.awk
#

BEGIN {
    stderr = "/dev/stderr";
}

{
    # accumulate MAPQ counts
    nMAPQ[$5]++ ;

    # count SAM records
    nSamRecords++ ;
}

END {
    printf( "Total lines processed: %d\n", NR ) > stderr;
    printf( "Total SAM records: %d\n", nSamRecords ) > stderr;

    # find minimum and maximum MAPQ
    minMAPQ = 999;
    maxMAPQ = 0;
    for( m in nMAPQ )
    {
        if( int(m) < int(minMAPQ) )
            minMAPQ = m;
        if( int(m) > int(maxMAPQ) )
            maxMAPQ = m;
    }

    printf( "----\n" ) > stderr;
    printf( "MAPQ\tn\n" ) > stderr;

    totalMAPQ = 0;
    for( m=int(minMAPQ); m<=int(maxMAPQ); ++m )
    {
        printf( "%4d\t%d\n", m, nMAPQ[m] ) > stderr;
        totalMAPQ += m * nMAPQ[m];
    }

    printf( "----\n" ) > stderr;
    printf( "average MAPQ: %5.2f\n", totalMAPQ/nSamRecords ) > stderr;
    printf( "average MAPQ (<%d only): %5.2f\n", \
            maxMAPQ, \
            (totalMAPQ-maxMAPQ*nMAPQ[maxMAPQ])/(nSamRecords-nMAPQ[maxMAPQ]) ) > stderr;

    printf( "Done.\n" ) > stderr;
}
```

Script S11. Distribution of MAPQ values in a SAM file.

```
#
# vcfx.awk
#
# Produce a distribution of two data fields in a specified VCF.
#
# Filters are specified as a semicolon-separated list of key-value pairs.  Filter operators are...
#  ==    QUERY.BD == FP                  exclude all FP calls
#  !=    QUERY.BD != TP                  exclude all non-TP calls
#  <     HG002.GQ < 20                   exclude calls with GQ less than 20
#  >     HG002.DP > 50                   exclude calls with DP greater than 50
#  :=    QUERY.BLT := het,hetalt,homalt   exclude calls with het, hetalt, or homalt genotypes
#
# Filters act by exclusion.  For example, 'QUERY.BVT != SNP' excludes all variant records
#  where the variant type is not SNP.
#
# Multiple filters are ORed together, i.e., a variant record is excluded if any filter condition is met.

function abend( _xc )
{
    exitCode = _xc;
    exit( _xc );
}

function round( _x,   ival, aval, frac )
{
    return int( (_x >= 0) ? _x+0.5 : _x-0.5 );
}

function trim( s )
{
    # trim spaces from both ends of the specified string
    sub( /[ ]+$/, "", s );
    sub( /^[ ]+/, "", s );
    return s;
}

function joinKeys( _a, _sep,   s, sepx, k )
{
    s = "";
    sepx = "";

    for( k in _a )
    {
        s = sprintf( "%s%s%s", s, sepx, k );   # (to join values we would use a[k] instead of k)
        sepx = _sep;
    }

    return s;
}

function opeq( a, b ) { return (a == b); }

function opne( a, b ) { return (a != b); }

function opgt( a, b ) { return (a > b); }

function oplt( a, b ) { return (a < b); }

function opin( a, b ) { return (a in b); }

function getAwkScriptFilename(    i, sarg )
{
    # look for the -f argument
    for( i=0; i<(length(PROCINFO["argv"])-1); i++ )
    {
        sarg = PROCINFO["argv"][i];
        if( sarg == "-f" )
            return PROCINFO["argv"][i+1];
```

```
    }

    # at this point we have not found the name of the awk script, so we just return "awk"
    return "awk";
}

function getValueINFO( _stub, _i,   a, fieldName, iField, af )
{
    split( $8, a, ";" );    # INFO name=value pairs

    # try to find the value at the most recently encountered field position
    fieldName = SYMTAB[_stub "fieldName"][_i];
    iField = SYMTAB[_stub "lastFieldIndex"][_i];
    split( a[iField], af, "=" );
    if( af[1] == fieldName )
        return af[2];

    # look for the field name in the INFO string
    for( iField in a )
    {
        split( a[iField], af, "=" );
        if( af[1] == fieldName )
        {
            SYMTAB[_stub "lastFieldIndex"][_i] = iField;
            return af[2];
        }
    }

    # at this point the field name is not in the INFO string
    SYMTAB[_stub "nMissing"][_i]++;
    return "?";
}

function getValueFORMAT( _stub, _i,   a, fieldName, iField, iCol )
{
    split( $9, a, ":" );    # FORMAT field names

    # try to find the value at the most recently encountered field position
    fieldName = SYMTAB[_stub "fieldName"][_i];
    iField = SYMTAB[_stub "lastFieldIndex"][_i];
    if( a[iField] == fieldName )
    {
        iCol = SYMTAB[_stub "columnIndex"][_i];
        split( $iCol, a, ":" );
        return a[iField];
    }

    # look for the field name in the FORMAT field
    for( iField in a )
    {
        if( a[iField] == fieldName )
        {
            SYMTAB[_stub "lastFieldIndex"][_i] = iField;
            iCol = SYMTAB[_stub "columnIndex"][_i];
            split( $iCol, a, ":" );
            return a[iField];
        }
    }

    # at this point the field name is not in the FORMAT field
    SYMTAB[_stub "nMissing"][_i]++;
    return "?";
}

function parseFieldsSpecification(    celf, cel, a, i, a2, sColumnName, sFieldName )
{
    if( length(fields) < 7 )
        return 0;
```

```
    # parse the field specifications
    celf = split( fields, a, ";" );
    for( i=1; i<=celf; ++i )
    {
        # separate the column ID from the field name
        cel = split( a[i], a2, "." );
        if( cel != 2 )
        {
            printf( "Invalid field specification: '%s'\n", a[i] ) > stderr;
            abend( 3 );
        }

        sColumnName = trim( a2[1] );
        sFieldName = trim( a2[2] );

        aVcolumnName[i] =     sColumnName;
        aVfieldName[i] =      sFieldName;
        aVcolumnIndex[i] =    0;
        aVfnGetValue[i] =     (sColumnName == "INFO") ? "getValueINFO" : "getValueFORMAT";
        aVofsValue[i] =       (sColumnName == "INFO") ? length(sFieldName) + 2 : 1;
        aVlastFieldIndex[i] = 0;
        aVnMissing[i] =       0;
        aVminValue[i] =       vLimit;
        aVmaxValue[i] =       -vLimit;
    }

    # return the number of fields
    return celf;
}

function parseFiltersSpecification(    ifn, cel, aff, i, aQ, cel2, a2, sColumnName, sFieldName, k, aK )
{
    # do nothing if there is no filter specification
    if( !filters )
    {
        delete aFcolumnName;
        return 0;
    }

    if( length(filters) < 5 )
    {
        printf( "Invalid filter specification: %s\n", filters ) > stderr;
        abend( 3 );
    }

    # We recognize a semicolon-separated list of filters, each specified as <key><op><value>:
    #
    #  <name> : as in FORMAT
    #  <op>   : < > <= >= == !=
    #  <value>: an awk string (which may, of course, be numerical according to the rules of awk)
    #
    # All filters are ORed together, e.g., to obtain the subset of TP variants with GQ >= 20:
    #  -v filters="QUERY.BD!=TP;QUERY.GQ<20"
    #
    # The same name can be specified more than once.
    #
    # Records with missing values are ignored unless specified explicitly, e.g.
    #  QUERY.GQ<20              includes all variants with GQ >= 20 or GQ = .
    #  QUERY.GQ<20;QUERY.GQ==.  includes all variants with GQ >= 20, excludes variants with missing GQ

    ifn["=="] = "opeq";
    ifn["!="] = "opne";
    ifn["<"]  = "oplt";
    ifn[">"]  = "opgt";
    ifn[":="] = "opin";

    cel = split( filters, aff, ";" );
    for( i=1; i<=cel; ++i )
    {
```

```
        # parse the ith field filter specification
        if( !match( aff[i], /([A-Za-z0-9\.]+)[ ]*([=>:<!]+)[ ]*(.+)/, aQ ) )
        {
            printf( "Invalid filter specification: %s\n", aff[i] ) > stderr;
            abend( 3 );
        }

        # separate the column ID from the field name
        cel2 = split( aQ[1], a2, "." );
        if( cel2 != 2 )
        {
            printf( "Invalid filter field specification in %s\n", aff[i] ) > stderr;
            abend( 3 );
        }

        sColumnName = trim( a2[1] );
        sFieldName = trim( a2[2] );

        aFcolumnName[i] =     sColumnName;
        aFfieldName[i] =      sFieldName;
        aFcolumnIndex[i] =    0;
        aFfnGetValue[i] =     (sColumnName == "INFO") ? "getValueINFO" : "getValueFORMAT";
        aFofsValue[i] =       (sColumnName == "INFO") ? length(sFieldName) + 2 : 1;
        aFlastFieldIndex[i] = 0;
        aFnMissing[i] =       0;
        aFop[i] =             aQ[2];
        aFfnop[i] =           ifn[aQ[2]];

        if( !(aFop[i]) || !(aFfnop[i]) )
        {
            printf( "Unrecognized operator in '%s': '%s'\n", aff[i], aQ[2] ) > stderr;
            abend( 2 );
        }

        if( aFfnop[i] == "opin" )
        {
            # build a list of comparison values
            split( aQ[3], aK, "," );
            for( k in aK )
                aFcmpValue[i][trim(aK[k])] = 1;
        }
        else
            aFcmpValue[i] = aQ[3];
    }

    # return the number of filters
    return cel;
}

function getColumnIndex( _stub, _i,   columnName, ic )
{
    columnName = SYMTAB[_stub "columnName"][_i];
    if( columnName == "INFO" )
        return;

    for( ic=10; ic<=NF; ++ic )
    {
        if( $ic == columnName )
        {
            SYMTAB[_stub "columnIndex"][_i] = ic;
            return;
        }
    }

    printf( "No sample column '%s' in VCF file\n", columnName ) > stderr;
    abend( 3 );
}

function agg1()
```

```
{
    # for comma-separated values, the rules of awk cause only the first value to be used
    #  (e.g., "39.3333,35.75" is counted as 39)
    binV[round(v[1])]++ ;
}

function emit1()
{
    printf( "%s.%s,n\n", aVcolumnName[1], aVfieldName[1] ) > csvFile;

    for( iv in binV )
        printf( "%d,%d\n", iv, binV[iv] ) > csvFile;
}

function agg2()
{
    binV[round(v[1])][round(v[2])]++ ;
}

function emit2(   iv1, iv2 )
{
    printf( "%s.%s,%s.%s,n\n", aVcolumnName[1], aVfieldName[1], aVcolumnName[2], aVfieldName[2] ) > csvFile;

    for( iv1 in binV )
    {
        for( iv2 in binV[iv1] )
            printf( "%d,%d,%d\n", iv1, iv2, binV[iv1][iv2] ) > csvFile;
    }
}

BEGIN {
    exitCode = 0;
    stderr = "/dev/stderr";
    vLimit = 99999999;

    nFields = parseFieldsSpecification();
    if( (nFields < 1) || (nFields > 2) )
    {
        printf( "The number of fields may only be 1 or 2\n" ) > stderr;
        abend( 4 );
    }

    # prepare to aggregate and emit counts
    fnAgg = "agg" nFields;
    fnEmit = "emit" nFields;

    parseFiltersSpecification();

    # traverse the VCF meta-information lines; reading them explicitly sets the FILENAME variable
    while( getline )
    {
        if( substr( $0, 1, 6 ) == "#CHROM" )
        {
            # ensure that the header line contains a FORMAT column and at least one sample ID
            if( NF < 10 )
            {
                printf( "VCF header does not contain at least one sample ID: %s\n", $0 ) > stderr;
                abend( 3 );
            }

            # save the index of each column
            for( i in aFcolumnName )
                getColumnIndex( "aF", i );

            for( i in aVcolumnName )
                getColumnIndex( "aV", i );

            # the header line is followed by the first variant record
            break;
```

```
        }
    }

    # echo the input parameters
    printf( "Starting %s...\n", getAwkScriptFilename() ) > stderr;
    printf( "input VCF: %s\n", FILENAME ) > stderr;

    sfx = "";
    sep = "";
    for( i in aVcolumnName )
    {
        sfx = sprintf( "%s%s%s.%s", sfx, sep, aVcolumnName[i], aVfieldName[i] );
        sep = ",";
    }
    printf( "fields   : %s\n", sfx ) > stderr;

    if( length(aFcolumnName) )
    {
        sfx = "";
        sep = "";
        for( i in aFcolumnName )
        {
            if( aFop[i] == ":=" )
                vx = joinKeys( aFcmpValue[i], "," );
            else
                vx = aFcmpValue[i];

            sfx = sprintf( "%s%s%s.%s %s %s", sfx, sep, aFcolumnName[i], aFfieldName[i], aFop[i], vx );
            sep = "; ";
        }
    }
    else
        sfx = "(none)";

    printf( "filter(s): %s\n", sfx ) > stderr;

    # prepare to track
    nRecords = 0;

    for( i=1; i<=nFields; ++i )
        v[i] = 0;
    delete binV;
}

{
    # filter the current variant record
    for( i in aFcolumnName )
    {
        fn = aFfnGetValue[i];
        fieldValue = trim( @fn( "aF", i ) );

        fn = aFfnop[i];
        if( @fn( fieldValue, aFcmpValue[i] ) )
            next;
    }

    # At this point we have a usable variant record.

    # get the field values
    for( iv in v )
    {
        fn = aVfnGetValue[iv];
        v[iv] = @fn( "aV", iv );

        if( v[iv] < aVminValue[iv] )
            aVminValue[iv] = v[iv];
        if( v[iv] > aVmaxValue[iv] )
            aVmaxValue[iv] = v[iv];
    }
```

```
    @fnAgg();
    ++nRecords;
}

END {
    if( exitCode )
        exit( exitCode );

    printf( "\nRecords counted: %d\n", nRecords ) > stderr;
    for( k in aFnMissing )
        printf( "Records missing %s.%s: %d\n", aFcolumnName[k], aFfieldName[k], aFnMissing[k] ) > stderr;
    for( k in aVnMissing )
        printf( "Records missing %s.%s: %d\n", aVcolumnName[k], aVfieldName[k], aVnMissing[k] ) > stderr;

    # emit counts as csv
    csvFile = "vcfx.csv";
    @fnEmit();

    printf( "Done.\n" ) > stderr;
}
```

Script S12. Variant annotation frequency distribution.

```
#
# doDiffGQ.sh
#

BCFTOOLSEXE=/path/to/bcftools-1.17/bcftools

echo "Start on $HOSTNAME at $(date +%FT%T)"

# echo the command tail
for arg; do echo " $"$((++n)): $arg; done

# sanity check
if [ ! -z "$1" ]
then
    echo 'Syntax: doDiffGQ.sh' >&2
    exit 1
fi

VCFHC=/path/to/HaplotypeCaller/HG002.TP.vcf.gz
VCFDV=/path/to/DeepVariant/HG002.TP.vcf.gz

VCFDIFF=${PWD}/diff

# create intersection and complements of DV and HC variant calls
bcftools isec ${VCFHC} ${VCFDV} -p ${VCFDIFF}

# At this point we have 4 VCF files in the diff subdirectory:
#  0000.vcf: HC only
#  0001.vcf: DV only
#  0002.vcf: HC records in both
#  0003.vcf: DV records in both

# get distribution of genotype field values
FIELDS='QUERY.GQ'
FILTERS='TRUTH.BD!=TP'
awk -f /path/to/vcfx.awk -v fields="${FIELDS}" -v filters="${FILTERS}" "${VCFDIFF}/0001.vcf"
cat vcfx.csv

echo 'Done.'
```

Script S13. GQ frequency distribution for true positive variant calls reported by DeepVariant but not by GATK HaplotypeCaller. Reads from HG002, aligned with Arioc to GRCh38.

Bowtie 2	2.4.5	https://bowtie-bio.sourceforge.net/bowtie2
BWA-MEM2	2.2.1	https://github.com/bwa-mem2/bwa-mem2
Arioc	1.51	https://github.com/RWilton/Arioc
HaplotypeCaller (GATK)	4.3.0.0	https://github.com/broadinstitute/gatk
DeepVariant	1.4.0	https://github.com/google/deepvariant
FreeBayes	1.3.6	https://github.com/freebayes/freebayes
hap.py	0.3.14	https://anaconda.org/bioconda/hap.py
samtools	1.17	https://www.htslib.org/download/
bcftools	1.17	https://www.htslib.org/download/
vcfeval	3.12.1	https://github.com/RealTimeGenomics/rtg-tools

Table T1. Software versions and download URLs.

	alignment scoring weights	minimum reportable alignment score	computational effort	paired-end mapping topology
Arioc	Wxmgs	Vt<br>Vtw	maxJ<br>seedDepth	pairFragmentLength<br>pairOrientation<br>pairCollision
Bowtie 2	ma<br>mp<br>rdg<br>rfg	score-min	-D<br>-R	minins<br>maxins<br>no-overlap<br>no-contain<br>dovetail
BWA-MEM	-A<br>-B<br>-O<br>-E	-T		-I

Table T2. Important runtime configuration parameters for general-purpose short-read aligners. Additional parameters that can be used to fine-tune alignment algorithms and to filter output content are not listed here.

HaplotypeCaller	`read-filter FragmentLengthReadFilter`<br>`min-fragment-length`<br>`minimum-mapping-quality`<br>`mapping-quality-threshold-for-genotyping`<br>`disable-cap-base-qualities-to-map-quality`
DeepVariant	`channels`<br>`min_base_quality`<br>`min_mapping_quality`
FreeBayes	`min-mapping-quality`<br>`use-mapping-quality`

Table T3. Important alignment-related runtime configuration parameters for germline variant callers. DeepVariant parameters are used in training the machine learning model. Additional parameters that can be used to fine-tune variant characterization and filter output content are not listed here.

	(pairs)		(truth)	TP	FP	recall	precision	F1	TiTv
a) min MAPQ = 20	10689816	INDEL	17445	17334	93	0.99364	0.99490	0.99427	
		SNP	108989	108388	217	0.99449	0.99800	0.99624	2.07913
b) min MAPQ = 27	10638504	INDEL	17445	17334	96	0.99364	0.99473	0.99419	
		SNP	108989	108387	200	0.99448	0.99816	0.99631	2.07906

Table T4. Effect of increased MAPQ threshold on variant calling accuracy. An increase in minimum MAPQ threshold from (a) 20 to (b) 27 results in a small increase in false positive indel calls but a decrease in false positive SNP calls.

Data from HG002 chr14 aligned to GRCh38.p14 with Arioc, variants called with GATK HaplotypeCaller.

a) Arioc

	truth	weights	sens	TP	FP	recall	precision	F1	TiTv
INDEL	17445	2, -4, -5, -3	81.7%	17332	106	0.99352	0.99419	0.99345	
		2, -6, -5, -3	81.4%	17334	103	0.99364	0.99435	0.99400	
		2, -8, -5, -3	81.0%	17334	93	0.99364	0.99490	0.99427	
		2, -14, -5, -3	80.4%	17335	72	0.99369	0.99605	0.99487	
		2, -20, -5, -3	80.0%	17329	81	0.99335	0.99555	0.99445	
SNP	108989	2, -4, -5, -3	81.7%	108395	304	0.99455	0.99720	0.99588	2.077
		2, -6, -5, -3	81.4%	108390	287	0.99450	0.99736	0.99593	2.078
		2, -8, -5, -3	81.0%	108388	217	0.99449	0.99800	0.99624	2.079
		2, -14, -5, -3	80.4%	108465	201	0.99519	0.99815	0.99667	2.078
		2, -20, -5, -3	80.0%	108412	156	0.99471	0.99856	0.99663	2.081

b) Bowtie 2

	truth	weights	sens	TP	FP	recall	precision	F1	TiTv
INDEL	17445	2, -6, -5, -3	78.9%	17265	101	0.98968	0.99444	0.99205	
		2, -8, -5, -3	78.2%	17262	106	0.98951	0.99416	0.99183	
		2, -14, -5, -3	76.8%	17221	117	0.98716	0.99354	0.99034	
		2, -20, -5, -3	76.6%	17213	121	0.98670	0.99332	0.99000	
SNP	108989	2, -6, -5, -3	78.9%	108390	192	0.99450	0.99823	0.99637	2.069
		2, -8, -5, -3	78.2%	108399	180	0.99459	0.99834	0.99646	2.075
		2, -14, -5, -3	76.8%	108344	218	0.99408	0.99799	0.99603	2.077
		2, -20, -5, -3	76.6%	108285	234	0.99354	0.99784	0.99569	2.071

(c) BWA-MEM

	truth	weights	sens	TP	FP	recall	precision	F1	TiTv
INDEL	17445	1, -4, -6, -1	82.4%	17331	93	0.99346	0.99490	0.99418	
		1, -6, -6, -1	82.2%	17334	101	0.99364	0.99446	0.99405	
		1, -9, -6, -1	81.9%	17336	96	0.99375	0.99473	0.99424	
		1, -12, -6, -1	81.8%	17334	97	0.99364	0.99468	0.99416	
SNP	108989	1, -4, -6, -1	82.4%	108381	259	0.99442	0.99762	0.99602	2.076
		1, -6, -6, -1	82.2%	108223	281	0.99297	0.99741	0.99519	2.080
		1, -9, -6, -1	81.9%	108233	297	0.99306	0.99726	0.99516	2.077
		1, -12, -6, -1	81.8%	108119	254	0.99202	0.99766	0.99483	2.073

Table T5. Effect of alignment scoring weights (match, mismatch, gap start, gap space) on alignment sensitivity (percentage of unduplicated properly mapped reads having MAPQ >= 4) and variant calling accuracy. Reads aligned with (a) Arioc, (b) Bowtie 2, and (c) BWA-MEM.

Aligner default scoring weights are highlighted.

With increasing mismatch penalties, all aligners report fewer properly mapped reads, but the number of FP variant calls may also decrease.

Data from HG002 chr14 aligned to GRCh38.p14, variants called with GATK HaplotypeCaller.

	GRCh37	GRCh38	T2T-CHM13
Arioc	0.936	0.956	0.978
BWA-MEM	0.970	0.975	0.984
Bowtie 2	0.892	0.914	0.932

Table T6. Fraction of reads having at least one proper mapping, using GRCh37, GRCh38, and T2T-CHM13.

Reads from HG002, without filtering on MAPQ or removal of duplicates.

	mismatches	GRCh37	GRCh38	CHM13
Arioc	0	0.722	0.725	0.739
	1	0.876	0.880	0.885
	2	0.922	0.926	0.929
	3	0.943	0.946	0.949
BWA-MEM	0	0.705	0.713	0.734
	1	0.850	0.860	0.875
	2	0.900	0.910	0.921
	3	0.923	0.933	0.942
Bowtie 2	0	0.743	0.739	0.760
	1	0.900	0.898	0.909
	2	0.947	0.945	0.952
	3	0.968	0.966	0.971

Table T7. Fraction of properly mapped reads having perfect or near-perfect alignment scores, using GRCh37, GRCh38, and T2T-CHM13.

Reads from HG002, without filtering on MAPQ or removal of duplicates. Cumulative fraction of properly mapped reads from AS distribution for each aligner and reference genome.

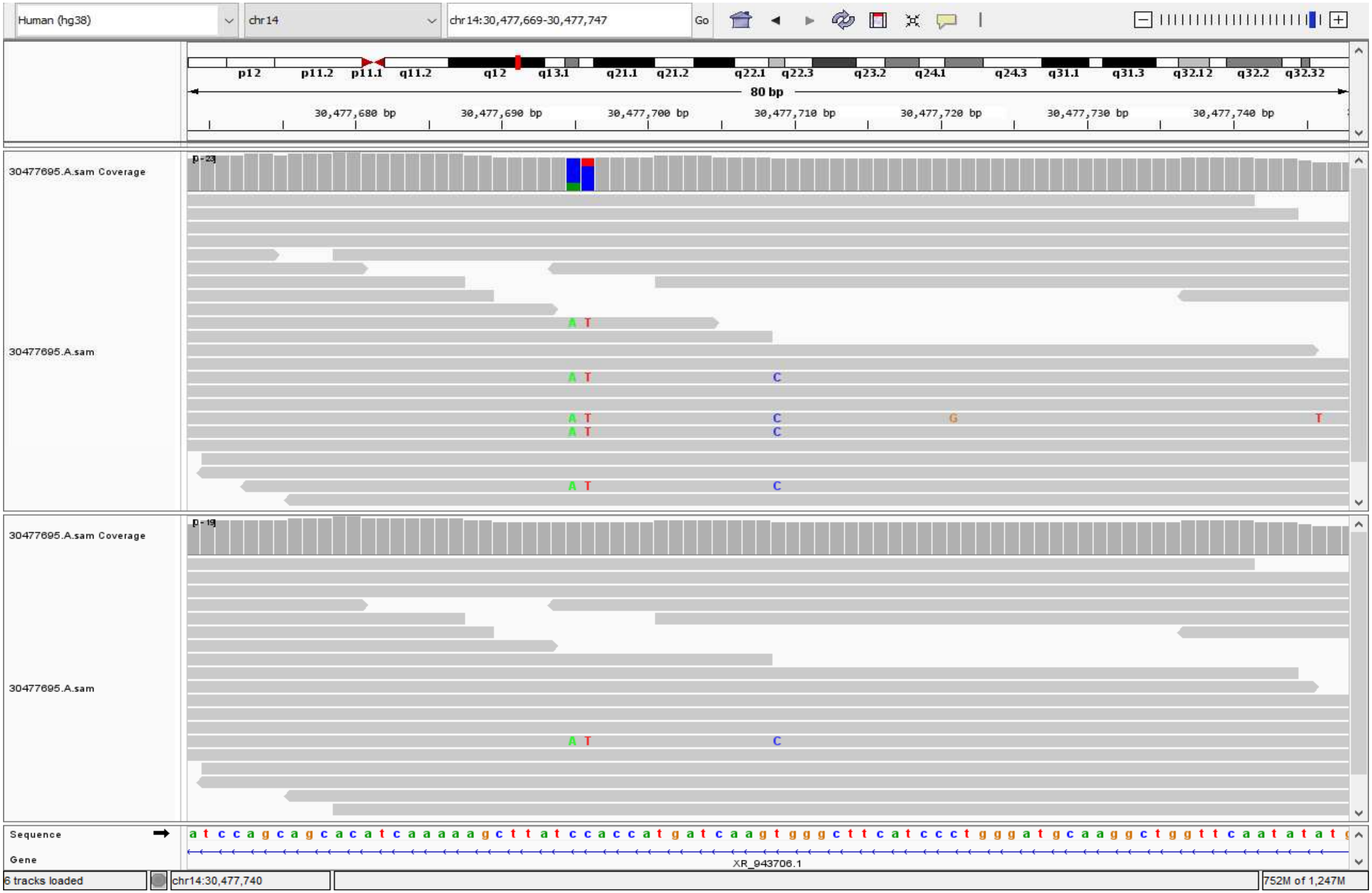

Figure F1. Alignment scoring weights can affect read mapping location.

Reads from HG002 mapped to GRCh38 chr14 using $W_m = 2$, $W_x = -6$, $W_g = -5$, $W_s = -3$ (upper track) and $W_m = 2$, $W_x = -8$, $W_g = -5$, $W_s = -3$ (lower track).

With $W_x = -6$, SNPs are identified at chr14:30,477,695 and chr14:30,477,696 due to the presence of an alternate allele in five different reads. With $W_x = -8$, the aligner mapped four of these five reads elsewhere in the reference genome with lower AS and lower MAPQ, thereby eliminating the SNP calls at these locations.

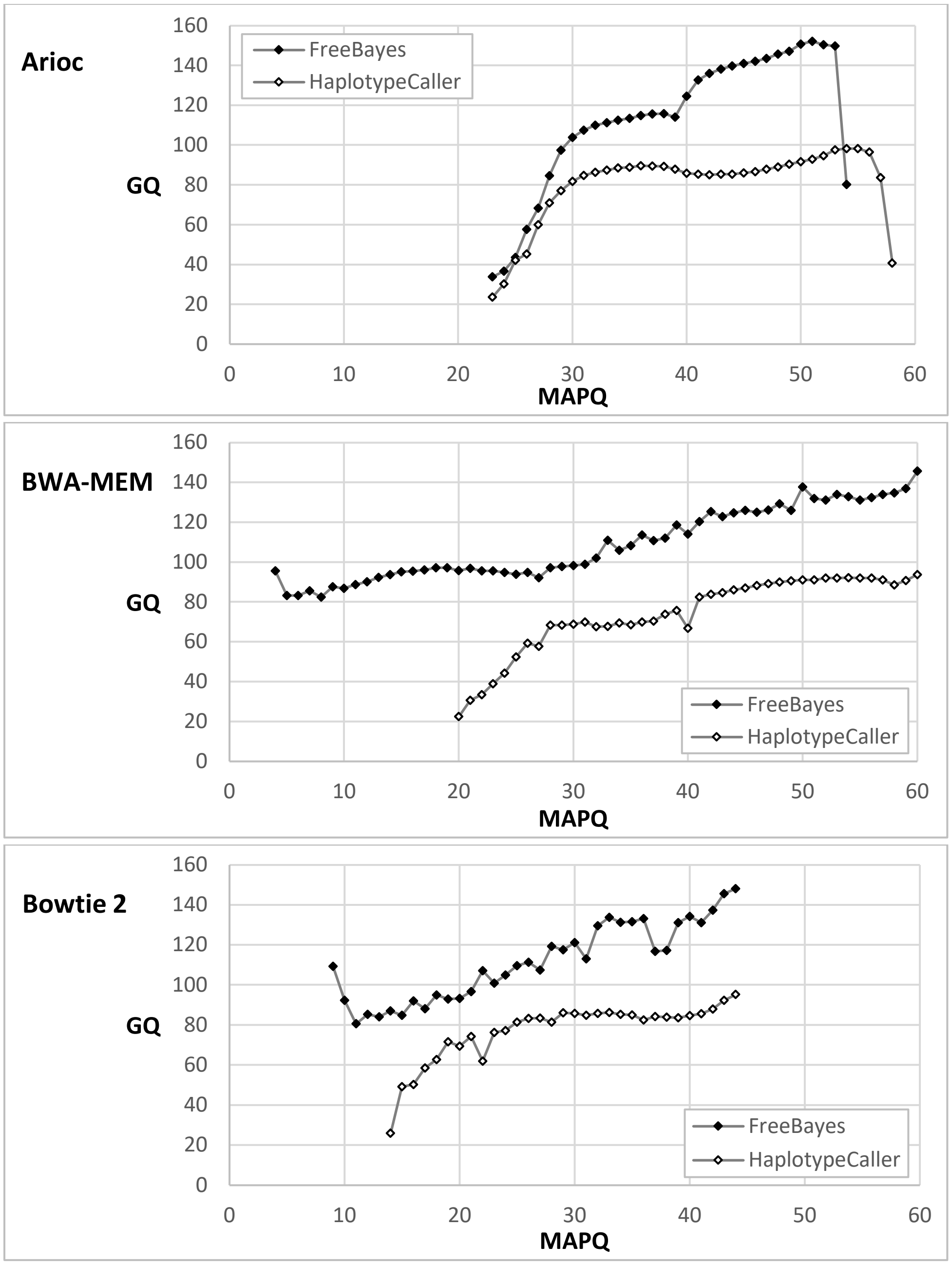

Figure F2. GQ (weighted average variant genotype quality) increases with higher MAPQ (read mapping quality). Reads from HG002 mapped to GRCh38.

HaplotypeCaller: minimum MAPQ = 20 (Arioc, BWA-MEM); minimum MAPQ = 14 (Bowtie 2). MAPQ from INFO.MQ (RMS mapping quality).

FreeBayes: minimum MAPQ = 1. MAPQ from INFO.MQM (mean mapping quality of observed alternate alleles).
